# Supplementary material for: Development of a detailed canine gait analysis method for evaluating harnesses: A pilot study
Source: PLoS One. 2022 Mar 9;17(3):e0264299. doi: 10.1371/journal.pone.0264299 (PMC8906618; doi:10.1371/journal.pone.0264299)
Supplement: S1 Table — Tabular results of the 53 calculated scalar parameters for all measurement scenarios. (PDF) [file pone.0264299.s001.pdf]

Spatio-temporal parameters (mean ± STD)

Case	Cycle Time [s]	Cadence [steps/min]	Back Stride Distance [m]	Front Stride Distance [m]	Speed [m/s]	BR Step Distance [m]	BR Step Height [mm]
Dog 1 reference	0.651 ± 0.030	369.5 ± 17.12	0.686 ± 0.033	0.686 ± 0.034	1.057 ± 0.081	0.324 ± 0.025	37.15 ± 2.557
Dog 1 K9 power	0.651 ± 0.027	369.4 ± 15.73	0.677 ± 0.029	0.676 ± 0.014	1.040 ± 0.042	0.316 ± 0.027	38.58 ± 3.801
Dog 1 K9 power (leash)	0.630 ± 0.024	381.5 ± 14.41	0.652 ± 0.021	0.649 ± 0.016	1.035 ± 0.052	0.325 ± 0.018	38.96 ± 1.649
Dog 1 K9 IDC	0.692 ± 0.021	347.3 ± 10.30	0.639 ± 0.036	0.637 ± 0.028	0.923 ± 0.050	0.287 ± 0.034	37.84 ± 2.076
Dog 1 K9 IDC (leash)	0.695 ± 0.032	346.2 ± 16.74	0.634 ± 0.035	0.636 ± 0.031	0.924 ± 0.068	0.312 ± 0.028	52.90 ± 12.232
Dog 1 K9 Duo-Flex	0.699 ± 0.027	344.1 ± 13.65	0.636 ± 0.037	0.638 ± 0.020	0.914 ± 0.053	0.304 ± 0.033	40.47 ± 4.078
Dog 1 K9 Duo-Flex (leash)	0.661 ± 0.042	364.5 ± 24.09	0.605 ± 0.046	0.606 ± 0.036	0.921 ± 0.098	0.292 ± 0.032	42.86 ± 3.341
Dog 2 reference	0.609 ± 0.049	396.4 ± 30.09	0.482 ± 0.040	0.482 ± 0.032	0.800 ± 0.061	0.204 ± 0.026	29.35 ± 2.769
Dog 2 K9 power	0.626 ± 0.039	384.6 ± 22.47	0.497 ± 0.032	0.496 ± 0.029	0.798 ± 0.071	0.216 ± 0.020	26.77 ± 2.324
Dog 2 K9 power (leash)	0.607 ± 0.056	398.9 ± 40.23	0.465 ± 0.026	0.464 ± 0.028	0.777 ± 0.102	0.191 ± 0.023	29.01 ± 2.854
Dog 2 K9 IDC	0.550 ± 0.045	439.6 ± 36.94	0.508 ± 0.046	0.506 ± 0.032	0.926 ± 0.059	0.210 ± 0.022	30.31 ± 4.699
Dog 2 K9 IDC (leash)	0.539 ± 0.042	448.1 ± 36.47	0.516 ± 0.039	0.514 ± 0.028	0.957 ± 0.120	0.216 ± 0.028	27.85 ± 1.673
Dog 2 K9 Duo-Flex	0.579 ± 0.033	416.0 ± 24.06	0.541 ± 0.040	0.541 ± 0.028	0.939 ± 0.088	0.241 ± 0.023	25.79 ± 1.866
Dog 2 K9 Duo-Flex (leash)	0.545 ± 0.027	441.5 ± 21.50	0.519 ± 0.029	0.520 ± 0.021	0.954 ± 0.050	0.222 ± 0.027	27.94 ± 2.730
Dog 3 reference	0.399 ± 0.028	604.7 ± 42.27	0.313 ± 0.014	0.313 ± 0.018	0.787 ± 0.075	0.146 ± 0.013	17.75 ± 1.939
Dog 3 K9 power	0.393 ± 0.034	614.4 ± 50.85	0.307 ± 0.013	0.308 ± 0.013	0.787 ± 0.079	0.144 ± 0.009	14.86 ± 3.073
Dog 3 K9 power (leash)	0.406 ± 0.028	594.4 ± 42.00	0.326 ± 0.010	0.322 ± 0.011	0.806 ± 0.063	0.158 ± 0.011	17.58 ± 2.932
Dog 3 K9 IDC	0.392 ± 0.026	614.5 ± 40.03	0.316 ± 0.023	0.314 ± 0.021	0.809 ± 0.087	0.147 ± 0.014	16.33 ± 2.557
Dog 3 K9 IDC (leash)	0.405 ± 0.036	597.2 ± 54.08	0.331 ± 0.017	0.329 ± 0.020	0.821 ± 0.072	0.161 ± 0.011	17.22 ± 2.459
Dog 4 reference	0.736 ± 0.034	327.0 ± 16.30	0.771 ± 0.042	0.773 ± 0.041	1.049 ± 0.021	0.391 ± 0.026	63.06 ± 2.568
Dog 4 K9 power	0.632 ± 0.021	380.1 ± 12.89	0.666 ± 0.030	0.666 ± 0.021	1.051 ± 0.023	0.345 ± 0.024	67.85 ± 1.591
Dog 4 K9 power (leash)	0.711 ± 0.016	337.6 ± 7.59	0.749 ± 0.019	0.748 ± 0.021	1.052 ± 0.022	0.375 ± 0.011	66.90 ± 2.276
Dog 4 K9 IDC	0.646 ± 0.034	372.5 ± 20.30	0.681 ± 0.038	0.680 ± 0.040	1.052 ± 0.037	0.349 ± 0.030	66.80 ± 3.866
Dog 4 K9 IDC (leash)	0.695 ± 0.016	345.3 ± 8.21	0.733 ± 0.015	0.729 ± 0.022	1.050 ± 0.017	0.367 ± 0.012	66.99 ± 1.894
Dog 4 K9 Duo-Flex	0.688 ± 0.032	349.9 ± 17.57	0.724 ± 0.037	0.720 ± 0.040	1.050 ± 0.013	0.360 ± 0.030	62.83 ± 2.985
Dog 4 K9 Duo-Flex (leash)	0.684 ± 0.017	351.2 ± 8.60	0.719 ± 0.022	0.719 ± 0.026	1.052 ± 0.021	0.362 ± 0.015	66.45 ± 3.681
Dog 4 Fressnapf	0.714 ± 0.032	336.8 ± 15.28	0.750 ± 0.034	0.749 ± 0.036	1.050 ± 0.028	0.383 ± 0.024	49.22 ± 4.154
Dog 4 Fressnapf (leash)	0.681 ± 0.029	353.1 ± 15.17	0.712 ± 0.041	0.711 ± 0.041	1.050 ± 0.032	0.369 ± 0.031	49.50 ± 4.161

Spatio-temporal parameters (mean ± STD)

Case	BR Swing Time [s]	BR Swing Ratio [%]	BR Stance Time [s]	BR Stance Ratio [%]	BR Foot Dist [m]	BL Step Distance [m]	BL Step Height [mm]
Dog 1 reference	0.239 ± 0.012	36.78 ± 1.969	0.412 ± 0.028	63.22 ± 1.969	0.732 ± 0.024	0.363 ± 0.027	33.26 ± 4.288
Dog 1 K9 power	0.239 ± 0.010	36.69 ± 1.841	0.412 ± 0.027	63.31 ± 1.841	0.728 ± 0.033	0.361 ± 0.022	33.15 ± 4.954
Dog 1 K9 power (leash)	0.218 ± 0.007	34.69 ± 1.428	0.412 ± 0.023	65.31 ± 1.428	0.756 ± 0.028	0.328 ± 0.016	33.95 ± 2.038
Dog 1 K9 IDC	0.241 ± 0.008	34.92 ± 1.453	0.450 ± 0.021	65.08 ± 1.453	0.703 ± 0.031	0.351 ± 0.018	34.86 ± 2.495
Dog 1 K9 IDC (leash)	0.224 ± 0.011	32.21 ± 1.752	0.471 ± 0.030	67.79 ± 1.752	0.773 ± 0.047	0.322 ± 0.027	49.78 ± 14.715
Dog 1 K9 Duo-Flex	0.235 ± 0.020	33.67 ± 2.690	0.463 ± 0.028	66.33 ± 2.690	0.724 ± 0.031	0.332 ± 0.031	37.18 ± 3.479
Dog 1 K9 Duo-Flex (leash)	0.214 ± 0.015	32.37 ± 1.977	0.447 ± 0.035	67.63 ± 1.977	0.742 ± 0.038	0.314 ± 0.027	35.86 ± 3.901
Dog 2 reference	0.193 ± 0.009	31.83 ± 2.328	0.416 ± 0.046	68.17 ± 2.328	0.591 ± 0.041	0.278 ± 0.024	30.54 ± 2.853
Dog 2 K9 power	0.195 ± 0.007	31.21 ± 2.102	0.431 ± 0.039	68.79 ± 2.102	0.601 ± 0.031	0.281 ± 0.019	30.72 ± 1.803
Dog 2 K9 power (leash)	0.185 ± 0.013	30.58 ± 2.388	0.422 ± 0.049	69.42 ± 2.388	0.599 ± 0.035	0.275 ± 0.022	31.41 ± 3.388
Dog 2 K9 IDC	0.181 ± 0.015	33.04 ± 1.609	0.368 ± 0.034	66.96 ± 1.609	0.596 ± 0.032	0.298 ± 0.028	34.08 ± 5.171
Dog 2 K9 IDC (leash)	0.176 ± 0.010	32.73 ± 2.413	0.363 ± 0.038	67.27 ± 2.413	0.607 ± 0.032	0.300 ± 0.020	29.06 ± 1.741
Dog 2 K9 Duo-Flex	0.197 ± 0.009	34.15 ± 2.328	0.382 ± 0.033	65.85 ± 2.328	0.619 ± 0.035	0.299 ± 0.022	27.44 ± 2.159
Dog 2 K9 Duo-Flex (leash)	0.179 ± 0.010	32.86 ± 1.469	0.366 ± 0.021	67.14 ± 1.469	0.599 ± 0.034	0.297 ± 0.016	27.39 ± 3.205
Dog 3 reference	0.139 ± 0.011	34.98 ± 3.928	0.260 ± 0.031	65.02 ± 3.928	0.347 ± 0.026	0.167 ± 0.009	19.49 ± 2.398
Dog 3 K9 power	0.140 ± 0.014	35.82 ± 4.089	0.253 ± 0.035	64.18 ± 4.089	0.335 ± 0.028	0.163 ± 0.011	16.77 ± 2.515
Dog 3 K9 power (leash)	0.134 ± 0.007	33.24 ± 2.663	0.271 ± 0.028	66.76 ± 2.663	0.385 ± 0.021	0.168 ± 0.006	19.33 ± 1.810
Dog 3 K9 IDC	0.139 ± 0.010	35.56 ± 3.464	0.253 ± 0.028	64.44 ± 3.464	0.337 ± 0.020	0.170 ± 0.013	18.27 ± 1.984
Dog 3 K9 IDC (leash)	0.131 ± 0.010	32.52 ± 2.474	0.274 ± 0.031	67.48 ± 2.474	0.393 ± 0.026	0.169 ± 0.009	20.53 ± 2.294
Dog 4 reference	0.248 ± 0.013	33.69 ± 0.963	0.488 ± 0.023	66.31 ± 0.963	0.881 ± 0.043	0.380 ± 0.022	66.70 ± 2.408
Dog 4 K9 power	0.206 ± 0.009	32.59 ± 1.208	0.426 ± 0.018	67.41 ± 1.208	0.783 ± 0.035	0.321 ± 0.018	70.50 ± 2.463
Dog 4 K9 power (leash)	0.230 ± 0.009	32.30 ± 0.886	0.482 ± 0.011	67.70 ± 0.886	0.899 ± 0.015	0.374 ± 0.014	70.02 ± 2.146
Dog 4 K9 IDC	0.213 ± 0.010	33.02 ± 1.756	0.433 ± 0.030	66.98 ± 1.756	0.792 ± 0.053	0.332 ± 0.027	69.13 ± 3.114
Dog 4 K9 IDC (leash)	0.221 ± 0.008	31.81 ± 0.832	0.474 ± 0.013	68.19 ± 0.832	0.884 ± 0.020	0.366 ± 0.012	69.63 ± 1.150
Dog 4 K9 Duo-Flex	0.227 ± 0.013	33.06 ± 0.934	0.460 ± 0.022	66.94 ± 0.934	0.836 ± 0.043	0.364 ± 0.020	65.45 ± 2.763
Dog 4 K9 Duo-Flex (leash)	0.225 ± 0.007	32.97 ± 0.767	0.458 ± 0.013	67.03 ± 0.767	0.856 ± 0.025	0.357 ± 0.017	70.11 ± 2.909
Dog 4 Fressnapf	0.236 ± 0.016	33.02 ± 1.406	0.478 ± 0.023	66.98 ± 1.406	0.875 ± 0.039	0.367 ± 0.020	48.05 ± 3.765
Dog 4 Fressnapf (leash)	0.221 ± 0.010	32.51 ± 1.322	0.460 ± 0.024	67.49 ± 1.322	0.863 ± 0.045	0.343 ± 0.035	49.64 ± 2.232

Spatio-temporal parameters (mean ± STD)

Case	BL Swing Time [s]	BL Swing Ratio [%]	BL Stance Time [s]	BL Stance Ratio [%]	BL Foot Dist [m]	FR Step Distance [m]	FR Step Height [mm]
Dog 1 reference	0.228 ± 0.015	35.06 ± 1.860	0.423 ± 0.024	64.94 ± 1.860	0.766 ± 0.027	0.338 ± 0.019	49.93 ± 6.672
Dog 1 K9 power	0.228 ± 0.016	35.00 ± 1.736	0.423 ± 0.020	65.00 ± 1.736	0.761 ± 0.021	0.334 ± 0.016	50.62 ± 7.899
Dog 1 K9 power (leash)	0.212 ± 0.004	33.64 ± 1.523	0.418 ± 0.025	66.36 ± 1.523	0.795 ± 0.018	0.336 ± 0.013	47.27 ± 2.733
Dog 1 K9 IDC	0.225 ± 0.013	32.57 ± 2.249	0.467 ± 0.026	67.43 ± 2.249	0.742 ± 0.022	0.312 ± 0.019	53.90 ± 7.743
Dog 1 K9 IDC (leash)	0.211 ± 0.009	30.46 ± 1.539	0.483 ± 0.030	69.54 ± 1.539	0.814 ± 0.041	0.317 ± 0.017	46.46 ± 3.414
Dog 1 K9 Duo-Flex	0.227 ± 0.018	32.46 ± 2.173	0.472 ± 0.023	67.54 ± 2.173	0.746 ± 0.024	0.321 ± 0.023	59.91 ± 11.720
Dog 1 K9 Duo-Flex (leash)	0.210 ± 0.012	31.82 ± 2.124	0.451 ± 0.039	68.18 ± 2.124	0.770 ± 0.027	0.314 ± 0.022	52.26 ± 8.133
Dog 2 reference	0.195 ± 0.009	32.21 ± 2.516	0.414 ± 0.047	67.79 ± 2.516	0.575 ± 0.019	0.225 ± 0.023	47.82 ± 5.496
Dog 2 K9 power	0.202 ± 0.008	32.37 ± 1.786	0.424 ± 0.036	67.63 ± 1.786	0.582 ± 0.018	0.233 ± 0.020	49.97 ± 3.857
Dog 2 K9 power (leash)	0.177 ± 0.012	29.24 ± 1.705	0.430 ± 0.046	70.76 ± 1.705	0.606 ± 0.020	0.208 ± 0.024	56.80 ± 8.652
Dog 2 K9 IDC	0.189 ± 0.010	34.57 ± 2.299	0.360 ± 0.040	65.43 ± 2.299	0.579 ± 0.020	0.240 ± 0.015	53.57 ± 3.434
Dog 2 K9 IDC (leash)	0.173 ± 0.010	32.23 ± 1.321	0.365 ± 0.034	67.77 ± 1.321	0.610 ± 0.024	0.230 ± 0.019	50.55 ± 4.858
Dog 2 K9 Duo-Flex	0.200 ± 0.008	34.64 ± 2.059	0.379 ± 0.031	65.36 ± 2.059	0.603 ± 0.019	0.251 ± 0.023	50.94 ± 3.996
Dog 2 K9 Duo-Flex (leash)	0.173 ± 0.010	31.88 ± 2.023	0.371 ± 0.026	68.12 ± 2.023	0.606 ± 0.016	0.225 ± 0.019	48.48 ± 3.680
Dog 3 reference	0.144 ± 0.013	36.18 ± 2.653	0.255 ± 0.023	63.82 ± 2.653	0.348 ± 0.017	0.155 ± 0.013	22.47 ± 3.904
Dog 3 K9 power	0.143 ± 0.012	36.49 ± 3.482	0.250 ± 0.032	63.51 ± 3.482	0.338 ± 0.024	0.158 ± 0.008	26.33 ± 3.659
Dog 3 K9 power (leash)	0.137 ± 0.007	33.83 ± 0.882	0.269 ± 0.021	66.17 ± 0.882	0.379 ± 0.023	0.165 ± 0.009	24.89 ± 2.093
Dog 3 K9 IDC	0.143 ± 0.010	36.48 ± 3.110	0.250 ± 0.026	63.52 ± 3.110	0.340 ± 0.021	0.165 ± 0.015	25.24 ± 5.068
Dog 3 K9 IDC (leash)	0.133 ± 0.016	32.81 ± 2.608	0.272 ± 0.026	67.19 ± 2.608	0.398 ± 0.022	0.171 ± 0.017	29.49 ± 7.335
Dog 4 reference	0.257 ± 0.014	34.87 ± 1.234	0.479 ± 0.025	65.13 ± 1.234	0.867 ± 0.041	0.372 ± 0.022	31.64 ± 5.093
Dog 4 K9 power	0.220 ± 0.008	34.87 ± 1.468	0.412 ± 0.021	65.13 ± 1.468	0.746 ± 0.027	0.320 ± 0.016	42.65 ± 4.653
Dog 4 K9 power (leash)	0.233 ± 0.007	32.81 ± 0.873	0.478 ± 0.013	67.19 ± 0.873	0.895 ± 0.019	0.361 ± 0.012	36.47 ± 4.158
Dog 4 K9 IDC	0.222 ± 0.014	34.38 ± 1.428	0.424 ± 0.025	65.62 ± 1.428	0.774 ± 0.037	0.352 ± 0.024	44.73 ± 12.064
Dog 4 K9 IDC (leash)	0.231 ± 0.010	33.22 ± 1.042	0.464 ± 0.012	66.78 ± 1.042	0.869 ± 0.018	0.372 ± 0.016	29.27 ± 1.973
Dog 4 K9 Duo-Flex	0.241 ± 0.014	35.06 ± 1.480	0.447 ± 0.025	64.94 ± 1.480	0.814 ± 0.037	0.349 ± 0.016	33.54 ± 4.905
Dog 4 K9 Duo-Flex (leash)	0.225 ± 0.008	32.86 ± 0.914	0.459 ± 0.014	67.14 ± 0.914	0.865 ± 0.026	0.330 ± 0.016	36.98 ± 3.785
Dog 4 Fressnapf	0.243 ± 0.014	34.09 ± 1.404	0.471 ± 0.024	65.91 ± 1.404	0.858 ± 0.038	0.366 ± 0.025	40.00 ± 9.629
Dog 4 Fressnapf (leash)	0.221 ± 0.016	32.52 ± 2.391	0.460 ± 0.029	67.48 ± 2.391	0.870 ± 0.030	0.352 ± 0.039	49.68 ± 10.307

Spatio-temporal parameters (mean ± STD)

Case	FR Swing Time [s]	FR Swing Ratio [%]	FR Stance Time [s]	FR Stance Ratio [%]	FR Foot Dist [m]	FL Step Distance [m]	FL Step Height [mm]
Dog 1 reference	-0.434 ± 0.023	-66.66 ± 2.430	1.085 ± 0.050	166.66 ± 2.430	0.814 ± 0.037	0.348 ± 0.023	54.575 ± 6.719
Dog 1 K9 power	-0.439 ± 0.024	-67.47 ± 2.354	1.090 ± 0.048	167.47 ± 2.354	0.815 ± 0.043	0.342 ± 0.010	55.462 ± 12.189
Dog 1 K9 power (leash)	-0.438 ± 0.024	-69.57 ± 2.822	1.068 ± 0.045	169.57 ± 2.822	0.827 ± 0.048	0.314 ± 0.011	44.714 ± 2.380
Dog 1 K9 IDC	-0.482 ± 0.028	-69.63 ± 3.105	1.173 ± 0.045	169.63 ± 3.105	0.778 ± 0.043	0.325 ± 0.017	56.609 ± 13.084
Dog 1 K9 IDC (leash)	-0.242 ± 0.355	-35.21 ± 50.858	0.937 ± 0.353	135.21 ± 50.858	0.801 ± 0.068	0.317 ± 0.021	43.961 ± 5.584
Dog 1 K9 Duo-Flex	-0.459 ± 0.132	-65.89 ± 18.558	1.158 ± 0.132	165.89 ± 18.558	0.784 ± 0.058	0.315 ± 0.027	60.846 ± 8.473
Dog 1 K9 Duo-Flex (leash)	-0.361 ± 0.242	-55.57 ± 35.914	1.022 ± 0.232	155.57 ± 35.914	0.777 ± 0.059	0.291 ± 0.029	46.754 ± 7.021
Dog 2 reference	0.148 ± 0.172	24.15 ± 28.738	0.461 ± 0.173	75.85 ± 28.738	0.568 ± 0.064	0.258 ± 0.020	36.957 ± 2.756
Dog 2 K9 power	0.031 ± 0.279	4.15 ± 45.376	0.596 ± 0.271	95.85 ± 45.376	0.592 ± 0.046	0.264 ± 0.016	35.866 ± 3.538
Dog 2 K9 power (leash)	0.022 ± 0.274	2.25 ± 46.225	0.585 ± 0.261	97.75 ± 46.225	0.606 ± 0.051	0.256 ± 0.015	41.452 ± 6.513
Dog 2 K9 IDC	-0.041 ± 0.276	-7.62 ± 50.390	0.591 ± 0.277	107.62 ± 50.390	0.583 ± 0.058	0.266 ± 0.022	40.896 ± 5.139
Dog 2 K9 IDC (leash)	0.093 ± 0.207	17.51 ± 38.178	0.446 ± 0.213	82.49 ± 38.178	0.611 ± 0.056	0.285 ± 0.017	38.659 ± 5.475
Dog 2 K9 Duo-Flex	-0.329 ± 0.178	-56.71 ± 30.514	0.908 ± 0.187	156.71 ± 30.514	0.621 ± 0.057	0.290 ± 0.018	41.413 ± 5.206
Dog 2 K9 Duo-Flex (leash)	-0.304 ± 0.167	-56.46 ± 29.972	0.848 ± 0.157	156.46 ± 29.972	0.591 ± 0.039	0.294 ± 0.014	41.538 ± 4.689
Dog 3 reference	0.122 ± 0.007	30.64 ± 2.967	0.277 ± 0.029	69.36 ± 2.967	0.394 ± 0.018	0.158 ± 0.010	25.906 ± 4.818
Dog 3 K9 power	0.121 ± 0.008	30.88 ± 3.195	0.273 ± 0.034	69.12 ± 3.195	0.393 ± 0.024	0.149 ± 0.012	23.231 ± 3.417
Dog 3 K9 power (leash)	0.120 ± 0.006	29.80 ± 2.352	0.285 ± 0.028	70.20 ± 2.352	0.416 ± 0.030	0.158 ± 0.005	25.792 ± 2.864
Dog 3 K9 IDC	0.117 ± 0.006	29.86 ± 2.316	0.275 ± 0.026	70.14 ± 2.316	0.393 ± 0.014	0.148 ± 0.017	23.133 ± 3.566
Dog 3 K9 IDC (leash)	0.123 ± 0.008	30.66 ± 3.058	0.282 ± 0.036	69.34 ± 3.058	0.417 ± 0.026	0.158 ± 0.007	30.148 ± 8.513
Dog 4 reference	-0.500 ± 0.023	-67.94 ± 1.552	1.235 ± 0.055	167.94 ± 1.552	0.945 ± 0.054	0.401 ± 0.025	37.643 ± 5.921
Dog 4 K9 power	-0.435 ± 0.018	-68.75 ± 2.088	1.067 ± 0.037	168.75 ± 2.088	0.822 ± 0.050	0.347 ± 0.018	50.176 ± 4.215
Dog 4 K9 power (leash)	-0.486 ± 0.016	-68.38 ± 2.336	1.198 ± 0.025	168.38 ± 2.336	0.946 ± 0.037	0.386 ± 0.014	45.491 ± 2.772
Dog 4 K9 IDC	-0.452 ± 0.035	-70.05 ± 5.100	1.098 ± 0.058	170.05 ± 5.100	0.840 ± 0.083	0.327 ± 0.023	77.811 ± 9.712
Dog 4 K9 IDC (leash)	-0.477 ± 0.017	-68.62 ± 1.289	1.173 ± 0.033	168.62 ± 1.289	0.919 ± 0.024	0.357 ± 0.010	65.819 ± 4.723
Dog 4 K9 Duo-Flex	-0.474 ± 0.030	-68.90 ± 2.421	1.161 ± 0.060	168.90 ± 2.421	0.887 ± 0.054	0.373 ± 0.032	44.544 ± 4.643
Dog 4 K9 Duo-Flex (leash)	-0.466 ± 0.014	-68.20 ± 1.553	1.150 ± 0.029	168.20 ± 1.553	0.870 ± 0.028	0.389 ± 0.015	45.636 ± 2.988
Dog 4 Fressnapf	-0.484 ± 0.084	-67.62 ± 11.373	1.198 ± 0.103	167.62 ± 11.373	0.895 ± 0.053	0.383 ± 0.022	44.439 ± 7.452
Dog 4 Fressnapf (leash)	-0.406 ± 0.190	-59.79 ± 27.447	1.087 ± 0.190	159.79 ± 27.447	0.862 ± 0.065	0.360 ± 0.020	45.901 ± 4.580

Spatio-temporal parameters (mean ± STD)

Case	FL Swing Time [s]	FL Swing Ratio [%]	FL Stance Time [s]	FL Stance Ratio [%]	FL Foot Dist [m]	Back Walking Base [m]	Front Walking Base [m]
Dog 1 reference	0.216 ± 0.009	33.14 ± 1.220	0.435 ± 0.025	66.86 ± 1.220	0.815 ± 0.024	147.89 ± 25.250	173.82 ± 17.550
Dog 1 K9 power	0.213 ± 0.011	32.71 ± 1.765	0.438 ± 0.026	67.29 ± 1.765	0.816 ± 0.031	146.53 ± 27.993	179.90 ± 18.187
Dog 1 K9 power (leash)	0.210 ± 0.007	33.36 ± 1.123	0.420 ± 0.022	66.64 ± 1.123	0.785 ± 0.019	164.06 ± 12.439	188.82 ± 12.055
Dog 1 K9 IDC	0.206 ± 0.014	29.79 ± 1.825	0.486 ± 0.019	70.21 ± 1.825	0.800 ± 0.022	140.79 ± 20.870	180.46 ± 16.460
Dog 1 K9 IDC (leash)	0.220 ± 0.011	31.76 ± 1.326	0.474 ± 0.026	68.24 ± 1.326	0.785 ± 0.032	175.70 ± 17.840	168.52 ± 10.382
Dog 1 K9 Duo-Flex	0.215 ± 0.010	30.87 ± 1.473	0.483 ± 0.025	69.13 ± 1.473	0.781 ± 0.037	170.25 ± 25.488	175.03 ± 28.013
Dog 1 K9 Duo-Flex (leash)	0.215 ± 0.011	32.68 ± 2.786	0.446 ± 0.043	67.32 ± 2.786	0.748 ± 0.031	179.21 ± 20.547	176.11 ± 14.006
Dog 2 reference	0.188 ± 0.008	31.01 ± 2.450	0.421 ± 0.048	68.99 ± 2.450	0.586 ± 0.051	170.13 ± 15.441	153.02 ± 16.766
Dog 2 K9 power	0.184 ± 0.008	29.52 ± 1.642	0.442 ± 0.036	70.48 ± 1.642	0.618 ± 0.034	165.44 ± 11.530	146.81 ± 11.679
Dog 2 K9 power (leash)	0.182 ± 0.016	30.17 ± 2.401	0.425 ± 0.048	69.83 ± 2.401	0.626 ± 0.027	204.28 ± 16.266	136.09 ± 15.685
Dog 2 K9 IDC	0.173 ± 0.009	31.62 ± 2.153	0.376 ± 0.041	68.38 ± 2.153	0.606 ± 0.043	163.59 ± 19.949	161.05 ± 12.216
Dog 2 K9 IDC (leash)	0.170 ± 0.012	31.54 ± 1.502	0.369 ± 0.033	68.46 ± 1.502	0.630 ± 0.022	181.07 ± 15.796	140.69 ± 18.034
Dog 2 K9 Duo-Flex	0.185 ± 0.010	32.05 ± 2.362	0.394 ± 0.033	67.95 ± 2.362	0.648 ± 0.031	158.28 ± 13.401	159.51 ± 13.867
Dog 2 K9 Duo-Flex (leash)	0.172 ± 0.006	31.60 ± 1.806	0.373 ± 0.026	68.40 ± 1.806	0.628 ± 0.034	175.16 ± 15.015	129.33 ± 21.657
Dog 3 reference	0.128 ± 0.008	32.15 ± 2.664	0.271 ± 0.027	67.85 ± 2.664	0.387 ± 0.016	72.72 ± 10.104	61.29 ± 11.752
Dog 3 K9 power	0.122 ± 0.005	31.25 ± 2.535	0.271 ± 0.032	68.75 ± 2.535	0.389 ± 0.021	71.56 ± 11.665	60.57 ± 11.845
Dog 3 K9 power (leash)	0.124 ± 0.005	30.67 ± 1.491	0.281 ± 0.025	69.33 ± 1.491	0.417 ± 0.016	75.20 ± 9.342	71.42 ± 9.294
Dog 3 K9 IDC	0.119 ± 0.008	30.54 ± 2.202	0.273 ± 0.024	69.46 ± 2.202	0.394 ± 0.017	71.13 ± 14.600	62.96 ± 17.783
Dog 3 K9 IDC (leash)	0.130 ± 0.007	32.28 ± 2.569	0.275 ± 0.033	67.72 ± 2.569	0.410 ± 0.028	94.96 ± 18.550	75.32 ± 9.704
Dog 4 reference	0.225 ± 0.012	30.63 ± 0.999	0.510 ± 0.025	69.37 ± 0.999	0.954 ± 0.047	132.38 ± 14.574	160.02 ± 18.892
Dog 4 K9 power	0.207 ± 0.009	32.69 ± 1.331	0.426 ± 0.019	67.31 ± 1.331	0.784 ± 0.032	127.11 ± 15.875	191.11 ± 14.891
Dog 4 K9 power (leash)	0.226 ± 0.009	31.79 ± 0.771	0.485 ± 0.010	68.21 ± 0.771	0.906 ± 0.021	149.85 ± 14.070	142.29 ± 15.145
Dog 4 K9 IDC	0.209 ± 0.011	32.39 ± 1.508	0.437 ± 0.029	67.61 ± 1.508	0.847 ± 0.039	149.29 ± 19.284	210.97 ± 17.779
Dog 4 K9 IDC (leash)	0.224 ± 0.008	32.24 ± 0.722	0.471 ± 0.011	67.76 ± 0.722	0.905 ± 0.016	153.13 ± 14.144	182.81 ± 23.010
Dog 4 K9 Duo-Flex	0.222 ± 0.014	32.23 ± 1.175	0.466 ± 0.023	67.77 ± 1.175	0.854 ± 0.045	134.29 ± 17.200	170.18 ± 21.250
Dog 4 K9 Duo-Flex (leash)	0.216 ± 0.005	31.65 ± 1.009	0.467 ± 0.017	68.35 ± 1.009	0.871 ± 0.032	156.29 ± 15.764	177.34 ± 19.097
Dog 4 Fressnapf	0.224 ± 0.015	31.40 ± 1.872	0.490 ± 0.029	68.60 ± 1.872	0.912 ± 0.051	130.99 ± 18.988	182.22 ± 16.912
Dog 4 Fressnapf (leash)	0.218 ± 0.012	32.09 ± 2.648	0.463 ± 0.034	67.91 ± 2.648	0.887 ± 0.073	158.61 ± 26.949	196.66 ± 19.804

Spatio-temporal parameters (mean ± STD)

Case	T1 hor. ROM [deg]	T13 hor. ROM [deg]	L7 hor. ROM [deg]	T1 sag. ROM [deg]	T13 sag. ROM [deg]	L7 sag. ROM [deg]	FR shoulder ROM [deg]
Dog 1 reference	9.03 ± 2.897	18.22 ± 2.038	20.11 ± 2.031	13.67 ± 5.576	9.02 ± 1.934	3.954 ± 1.063	24.97 ± 4.268
Dog 1 K9 power	8.82 ± 3.914	20.54 ± 2.620	19.23 ± 1.191	12.82 ± 6.010	9.55 ± 1.870	4.684 ± 1.204	37.11 ± 3.995
Dog 1 K9 power (leash)	9.35 ± 3.293	30.18 ± 4.140	24.04 ± 1.618	7.95 ± 3.742	8.36 ± 1.327	5.229 ± 0.910	34.03 ± 1.158
Dog 1 K9 IDC	9.04 ± 3.259	24.76 ± 3.357	19.91 ± 2.116	12.86 ± 5.621	9.83 ± 1.849	3.769 ± 0.810	40.56 ± 4.117
Dog 1 K9 IDC (leash)	10.56 ± 5.753	29.93 ± 3.198	21.70 ± 1.560	8.42 ± 3.278	8.96 ± 1.119	5.485 ± 1.165	38.49 ± 3.482
Dog 1 K9 Duo-Flex	9.05 ± 2.692	21.60 ± 2.511	20.13 ± 2.292	14.51 ± 5.429	9.08 ± 2.858	4.030 ± 1.143	62.26 ± 5.294
Dog 1 K9 Duo-Flex (leash)	11.00 ± 7.212	26.93 ± 4.453	23.55 ± 2.316	11.47 ± 5.105	6.97 ± 1.607	4.643 ± 1.090	43.96 ± 3.167
Dog 2 reference	7.41 ± 3.113	13.12 ± 1.709	12.00 ± 1.470	13.63 ± 5.772	6.67 ± 1.191	4.393 ± 0.859	31.00 ± 2.595
Dog 2 K9 power	11.03 ± 3.294	33.33 ± 3.272	15.31 ± 2.987	7.44 ± 4.231	10.33 ± 2.464	4.075 ± 0.703	30.63 ± 1.349
Dog 2 K9 power (leash)	8.51 ± 2.162	13.69 ± 1.545	0.00 ± 0.000	7.00 ± 3.185	6.36 ± 1.123	0.000 ± 0.000	30.61 ± 2.020
Dog 2 K9 IDC	9.32 ± 2.644	23.37 ± 2.499	13.10 ± 1.426	9.09 ± 5.420	7.63 ± 2.120	3.797 ± 0.951	38.71 ± 2.175
Dog 2 K9 IDC (leash)	12.85 ± 6.724	25.36 ± 4.334	16.40 ± 3.291	8.93 ± 4.934	8.42 ± 2.061	5.122 ± 0.708	41.20 ± 2.469
Dog 2 K9 Duo-Flex	11.78 ± 4.497	19.69 ± 2.804	12.08 ± 1.458	13.02 ± 6.172	9.30 ± 2.073	4.139 ± 0.757	31.46 ± 3.547
Dog 2 K9 Duo-Flex (leash)	11.52 ± 5.895	27.39 ± 3.916	18.33 ± 2.479	8.53 ± 3.944	6.00 ± 1.625	2.383 ± 0.667	30.54 ± 1.500
Dog 3 reference	13.72 ± 3.303	16.39 ± 1.763	18.68 ± 2.104	15.42 ± 6.410	10.95 ± 3.503	3.393 ± 0.999	24.31 ± 3.332
Dog 3 K9 power	13.91 ± 1.799	18.84 ± 2.381	17.68 ± 2.229	15.15 ± 5.332	10.68 ± 3.104	3.589 ± 1.010	28.26 ± 2.466
Dog 3 K9 power (leash)	13.11 ± 2.588	23.19 ± 1.066	0.00 ± 0.000	12.35 ± 2.561	4.29 ± 1.181	0.000 ± 0.000	31.97 ± 1.228
Dog 3 K9 IDC	12.26 ± 2.744	22.15 ± 4.038	20.25 ± 1.777	15.69 ± 6.388	9.69 ± 2.398	3.420 ± 1.436	31.11 ± 5.186
Dog 3 K9 IDC (leash)	17.88 ± 3.300	19.18 ± 4.641	25.97 ± 4.295	16.35 ± 6.313	7.81 ± 2.379	2.967 ± 1.120	39.59 ± 4.344
Dog 4 reference	20.46 ± 2.638	22.84 ± 1.456	16.28 ± 1.555	10.57 ± 3.970	8.16 ± 1.168	3.498 ± 0.676	28.46 ± 1.794
Dog 4 K9 power	11.40 ± 2.021	19.47 ± 2.313	17.04 ± 1.354	13.89 ± 5.804	8.97 ± 1.490	3.295 ± 0.529	42.07 ± 2.470
Dog 4 K9 power (leash)	12.71 ± 2.221	19.22 ± 1.431	16.83 ± 1.145	12.93 ± 4.202	6.42 ± 0.976	4.112 ± 0.686	40.51 ± 1.513
Dog 4 K9 IDC	12.64 ± 2.558	19.58 ± 1.614	16.88 ± 1.725	8.83 ± 4.135	7.64 ± 1.215	3.848 ± 1.128	34.18 ± 1.911
Dog 4 K9 IDC (leash)	14.75 ± 1.142	18.46 ± 0.941	18.06 ± 1.057	10.37 ± 3.306	7.63 ± 1.110	3.965 ± 0.645	35.95 ± 2.112
Dog 4 K9 Duo-Flex	18.99 ± 4.615	22.34 ± 2.192	14.58 ± 1.254	9.94 ± 3.231	8.32 ± 1.035	3.844 ± 0.600	37.91 ± 2.794
Dog 4 K9 Duo-Flex (leash)	14.01 ± 1.890	22.55 ± 1.578	14.24 ± 1.418	11.12 ± 2.813	5.90 ± 0.982	4.088 ± 0.603	32.13 ± 2.184
Dog 4 Fressnapf	11.91 ± 3.701	17.56 ± 2.383	15.32 ± 1.223	14.09 ± 5.454	9.67 ± 2.726	3.005 ± 0.655	25.89 ± 2.349
Dog 4 Fressnapf (leash)	11.79 ± 2.867	15.18 ± 2.183	16.22 ± 1.647	16.79 ± 6.831	7.91 ± 1.413	3.185 ± 0.652	33.47 ± 3.500

Spatio-temporal parameters (mean ± STD)

Case	FL shoulder ROM [deg]	FR elbow ROM [deg]	FL elbow ROM [deg]	FR carpal ROM [deg]	FL carpal ROM [deg]	BR hip ROM [deg]	BL hip ROM [deg]
Dog 1 reference	29.13 ± 2.296	44.44 ± 3.850	46.39 ± 2.787	79.28 ± 4.451	86.0 ± 5.91	33.84 ± 2.451	31.45 ± 2.188
Dog 1 K9 power	43.19 ± 1.977	47.57 ± 5.379	50.31 ± 3.933	84.24 ± 4.085	87.7 ± 5.71	33.44 ± 2.661	29.66 ± 2.377
Dog 1 K9 power (leash)	45.60 ± 1.613	47.61 ± 2.640	50.32 ± 4.726	89.92 ± 3.527	83.9 ± 1.98	37.78 ± 1.328	36.88 ± 2.377
Dog 1 K9 IDC	39.29 ± 3.657	44.52 ± 3.609	54.00 ± 4.450	81.28 ± 5.381	88.0 ± 6.04	30.90 ± 2.066	29.04 ± 2.523
Dog 1 K9 IDC (leash)	37.79 ± 4.268	43.18 ± 5.081	45.48 ± 3.981	78.39 ± 4.412	75.6 ± 6.14	37.33 ± 3.450	34.79 ± 3.219
Dog 1 K9 Duo-Flex	55.38 ± 3.423	41.36 ± 5.424	43.62 ± 7.600	78.76 ± 6.381	81.7 ± 8.21	33.11 ± 2.918	30.92 ± 2.690
Dog 1 K9 Duo-Flex (leash)	45.84 ± 4.071	45.95 ± 4.157	46.36 ± 3.365	79.62 ± 5.078	77.8 ± 4.61	41.15 ± 2.798	38.44 ± 3.269
Dog 2 reference	26.66 ± 1.574	48.22 ± 7.400	46.94 ± 5.843	45.25 ± 4.529	64.5 ± 7.44	20.24 ± 2.726	17.70 ± 3.550
Dog 2 K9 power	27.31 ± 1.575	51.23 ± 5.644	49.53 ± 3.477	50.03 ± 3.588	71.7 ± 3.70	24.55 ± 3.061	18.78 ± 2.837
Dog 2 K9 power (leash)	29.45 ± 2.504	57.41 ± 2.957	58.67 ± 4.348	57.45 ± 3.381	79.6 ± 2.88	33.06 ± 2.220	26.13 ± 3.350
Dog 2 K9 IDC	31.92 ± 2.284	50.46 ± 3.220	49.94 ± 4.669	47.64 ± 6.941	70.6 ± 6.92	21.99 ± 2.472	15.68 ± 4.170
Dog 2 K9 IDC (leash)	35.18 ± 2.112	52.35 ± 6.466	53.94 ± 4.739	53.83 ± 2.512	76.4 ± 3.74	30.11 ± 2.971	20.25 ± 3.347
Dog 2 K9 Duo-Flex	33.09 ± 1.571	55.54 ± 3.911	55.92 ± 3.720	52.37 ± 3.194	76.1 ± 4.55	25.18 ± 2.446	17.96 ± 3.956
Dog 2 K9 Duo-Flex (leash)	30.03 ± 1.825	56.69 ± 2.645	58.85 ± 4.322	55.46 ± 3.872	76.3 ± 3.94	33.78 ± 3.255	28.13 ± 2.261
Dog 3 reference	20.85 ± 2.299	62.28 ± 4.371	62.93 ± 4.611	134.81 ± 12.192	121.1 ± 8.71	23.00 ± 2.877	21.14 ± 2.452
Dog 3 K9 power	27.65 ± 1.705	63.60 ± 4.068	63.29 ± 3.847	138.43 ± 14.845	124.6 ± 7.71	22.46 ± 2.304	19.08 ± 2.376
Dog 3 K9 power (leash)	31.56 ± 1.936	66.31 ± 2.223	67.80 ± 2.003	133.59 ± 3.512	119.1 ± 7.52	29.52 ± 1.666	26.74 ± 2.857
Dog 3 K9 IDC	36.80 ± 5.707	60.57 ± 5.405	59.27 ± 5.485	136.99 ± 19.665	128.3 ± 10.23	23.83 ± 2.883	20.92 ± 2.116
Dog 3 K9 IDC (leash)	36.89 ± 4.604	69.45 ± 5.038	64.88 ± 4.430	126.00 ± 12.934	115.8 ± 8.61	31.45 ± 2.800	32.61 ± 3.427
Dog 4 reference	25.91 ± 1.740	52.54 ± 2.626	61.01 ± 3.164	99.70 ± 3.116	106.8 ± 3.17	27.38 ± 1.025	28.71 ± 1.209
Dog 4 K9 power	39.14 ± 3.479	54.08 ± 3.700	53.42 ± 3.599	100.63 ± 3.761	104.9 ± 3.15	28.19 ± 1.460	27.64 ± 1.511
Dog 4 K9 power (leash)	40.38 ± 1.964	52.81 ± 2.258	52.33 ± 2.514	103.38 ± 3.573	100.0 ± 2.73	30.96 ± 0.629	33.26 ± 0.860
Dog 4 K9 IDC	36.33 ± 3.605	55.34 ± 5.488	61.11 ± 4.507	109.30 ± 4.897	100.9 ± 3.42	26.56 ± 2.256	27.66 ± 2.065
Dog 4 K9 IDC (leash)	34.80 ± 1.600	55.64 ± 2.721	56.98 ± 2.718	105.04 ± 4.364	97.1 ± 2.24	30.53 ± 0.629	32.37 ± 1.029
Dog 4 K9 Duo-Flex	34.03 ± 1.605	50.30 ± 2.431	49.15 ± 2.357	99.43 ± 3.489	104.4 ± 3.53	27.67 ± 1.580	28.97 ± 1.989
Dog 4 K9 Duo-Flex (leash)	28.37 ± 1.290	49.97 ± 2.108	53.58 ± 2.282	89.20 ± 3.185	99.0 ± 2.50	29.88 ± 0.649	33.49 ± 1.132
Dog 4 Fressnapf	22.66 ± 2.114	55.37 ± 5.243	53.29 ± 3.857	96.18 ± 4.828	108.4 ± 4.05	30.08 ± 2.249	35.40 ± 2.462
Dog 4 Fressnapf (leash)	29.47 ± 2.542	55.60 ± 5.722	54.70 ± 4.438	90.83 ± 6.601	102.0 ± 8.25	33.70 ± 4.011	40.21 ± 2.560

Spatio-temporal parameters (mean ± STD)

Case	BR stifle ROM [deg]	BL stifle ROM [deg]	BR hock ROM [deg]	BL hock ROM [deg]
Dog 1 reference	39.40 ± 2.264	39.39 ± 3.411	36.14 ± 1.822	36.47 ± 3.314
Dog 1 K9 power	40.78 ± 3.478	38.53 ± 4.347	36.27 ± 2.994	34.92 ± 3.269
Dog 1 K9 power (leash)	39.79 ± 2.851	37.36 ± 3.228	41.37 ± 2.867	39.98 ± 3.643
Dog 1 K9 IDC	39.97 ± 1.557	39.67 ± 2.145	35.58 ± 2.286	32.73 ± 4.167
Dog 1 K9 IDC (leash)	50.22 ± 8.283	50.76 ± 11.144	45.98 ± 11.258	45.89 ± 12.379
Dog 1 K9 Duo-Flex	39.81 ± 3.224	39.01 ± 2.749	36.27 ± 4.253	35.32 ± 4.478
Dog 1 K9 Duo-Flex (leash)	38.34 ± 3.999	34.53 ± 4.161	46.47 ± 4.126	41.17 ± 3.719
Dog 2 reference	48.01 ± 5.276	44.07 ± 2.569	32.13 ± 5.000	27.97 ± 3.229
Dog 2 K9 power	44.87 ± 3.596	44.18 ± 3.365	31.68 ± 3.222	30.48 ± 2.705
Dog 2 K9 power (leash)	39.11 ± 1.784	43.61 ± 2.449	38.59 ± 3.128	43.42 ± 3.903
Dog 2 K9 IDC	47.05 ± 4.026	46.50 ± 3.148	36.99 ± 2.976	31.48 ± 2.500
Dog 2 K9 IDC (leash)	43.48 ± 2.436	45.48 ± 3.047	36.42 ± 5.862	36.14 ± 3.586
Dog 2 K9 Duo-Flex	45.01 ± 3.246	43.05 ± 2.905	38.49 ± 3.250	32.43 ± 4.044
Dog 2 K9 Duo-Flex (leash)	41.67 ± 1.676	43.31 ± 1.881	41.13 ± 3.441	39.54 ± 3.187
Dog 3 reference	54.32 ± 3.289	56.08 ± 2.631	38.10 ± 3.797	34.31 ± 3.263
Dog 3 K9 power	50.88 ± 3.234	53.63 ± 2.267	36.40 ± 3.620	34.86 ± 1.978
Dog 3 K9 power (leash)	51.43 ± 2.852	53.16 ± 2.422	39.09 ± 2.412	32.40 ± 1.743
Dog 3 K9 IDC	51.92 ± 3.104	53.73 ± 2.444	35.80 ± 3.839	35.72 ± 3.824
Dog 3 K9 IDC (leash)	50.57 ± 3.979	49.82 ± 4.599	46.82 ± 9.439	40.43 ± 7.132
Dog 4 reference	39.74 ± 2.471	40.76 ± 1.777	32.67 ± 1.725	36.64 ± 2.179
Dog 4 K9 power	45.39 ± 2.007	44.18 ± 2.018	31.86 ± 2.355	34.45 ± 1.785
Dog 4 K9 power (leash)	40.51 ± 1.099	42.70 ± 1.987	36.20 ± 2.315	42.40 ± 2.154
Dog 4 K9 IDC	46.64 ± 3.700	44.21 ± 3.804	31.65 ± 2.471	34.86 ± 2.375
Dog 4 K9 IDC (leash)	40.97 ± 2.050	41.00 ± 1.628	34.52 ± 2.362	40.54 ± 1.848
Dog 4 K9 Duo-Flex	42.09 ± 2.442	41.36 ± 2.145	31.58 ± 2.190	35.57 ± 2.671
Dog 4 K9 Duo-Flex (leash)	39.11 ± 2.469	43.30 ± 1.804	32.60 ± 1.834	42.50 ± 2.401
Dog 4 Fressnapf	44.62 ± 2.581	41.63 ± 2.331	33.38 ± 2.561	38.30 ± 2.722
Dog 4 Fressnapf (leash)	39.62 ± 3.711	39.71 ± 3.232	33.64 ± 5.003	41.68 ± 5.093
