# Supplementary material for: Development of a detailed canine gait analysis method for evaluating harnesses: A pilot study
Source: PLoS One. 2022 Mar 9;17(3):e0264299. doi: 10.1371/journal.pone.0264299 (PMC8906618; doi:10.1371/journal.pone.0264299)
Supplement: S2 Table — Tabular results of the p-values of the Anderson-Darling normality test for the 53 calculated scalar parameters for all measurement scenarios. (PDF) [file pone.0264299.s002.pdf]

Anderson-Darling normality test results

Case	Cycle Time	Cadence	Back Stride Distance	Front Stride Distance	Speed	BR Step Distance	BR Step Height	BR Swing Time	BR Swing Ratio	BR Stance Time	BR Stance Ratio
Dog 1 reference	0.4346	0.3910	0.2121	0.1041	0.1816	0.7171	0.3117	0.0023	0.7138	0.1218	0.7138
Dog 1 K9 power	0.2524	0.1922	0.1307	0.2656	0.4515	0.1581	0.0088	0.0073	0.0719	0.0431	0.0719
Dog 1 K9 power (leash)	0.0301	0.0429	0.5341	0.9317	0.3534	0.5888	0.3489	0.0005	0.0351	0.1802	0.0351
Dog 1 K9 IDC	0.6197	0.5973	0.5436	0.2002	0.5313	0.1519	0.0574	0.0010	0.8815	0.3647	0.8815
Dog 1 K9 IDC (leash)	0.0450	0.0243	0.1019	0.5875	0.7435	0.1376	0.0557	0.0311	0.5284	0.3252	0.5284
Dog 1 K9 Duo-Flex	0.1973	0.1084	0.0283	0.1083	0.1297	0.0940	0.0177	0.0543	0.7362	0.6194	0.7362
Dog 1 K9 Duo-Flex (leash)	0.3238	0.0891	0.5941	0.6024	0.2403	0.9767	0.7323	0.0590	0.0369	0.3956	0.0369
Dog 2 reference	0.0737	0.3306	0.0583	0.3652	0.4723	0.8649	0.2206	0.0077	0.2218	0.0187	0.2218
Dog 2 K9 power	0.0284	0.1846	0.3276	0.3173	0.1553	0.8455	0.0754	0.0005	0.7991	0.0719	0.7991
Dog 2 K9 power (leash)	0.0368	0.0039	0.5036	0.0403	0.0005	0.9701	0.8122	0.4075	0.1517	0.0295	0.1517
Dog 2 K9 IDC	0.0008	0.0005	0.0166	0.4650	0.0621	0.8017	0.0116	0.0265	0.4251	0.0237	0.4251
Dog 2 K9 IDC (leash)	0.2264	0.0975	0.8599	0.7011	0.7091	0.4949	0.6118	0.1043	0.4179	0.5287	0.4179
Dog 2 K9 Duo-Flex	0.1133	0.1074	0.3403	0.9658	0.8220	0.6225	0.6159	0.0130	0.5550	0.2028	0.5550
Dog 2 K9 Duo-Flex (leash)	0.1700	0.1198	0.0006	0.0827	0.6874	0.0307	0.0195	0.0151	0.0438	0.0152	0.0438
Dog 3 reference	0.1399	0.2005	0.0576	0.3239	0.6267	0.2905	0.1509	0.0012	0.2013	0.6164	0.2013
Dog 3 K9 power	0.3072	0.5193	0.2489	0.4369	0.8330	0.7883	0.6009	0.0005	0.4892	0.0329	0.4892
Dog 3 K9 power (leash)	0.5685	0.4981	0.6007	0.2376	0.4076	0.4159	0.0242	0.0628	0.8885	0.5183	0.8885
Dog 3 K9 IDC	0.2875	0.3169	0.0378	0.0949	0.6694	0.6244	0.1109	0.0015	0.2852	0.3438	0.2852
Dog 3 K9 IDC (leash)	0.0601	0.0242	0.7585	0.6005	0.2392	0.1281	0.2280	0.0013	0.0978	0.5497	0.0978
Dog 4 reference	0.0005	0.0005	0.0005	0.0005	0.4912	0.0005	0.1344	0.0005	0.0697	0.0005	0.0697
Dog 4 K9 power	0.1402	0.1454	0.7478	0.7937	0.4543	0.0411	0.3347	0.1971	0.2952	0.3298	0.2952
Dog 4 K9 power (leash)	0.0962	0.0929	0.0670	0.4579	0.0624	0.1373	0.2158	0.0731	0.7859	0.2579	0.7859
Dog 4 K9 IDC	0.5255	0.2060	0.4664	0.3026	0.8331	0.8364	0.0125	0.0160	0.0220	0.1364	0.0220
Dog 4 K9 IDC (leash)	0.2961	0.2705	0.3159	0.6316	0.2095	0.4184	0.9081	0.0109	0.1019	0.2734	0.1019
Dog 4 K9 Duo-Flex	0.0790	0.0253	0.3015	0.0743	0.6064	0.0178	0.8677	0.0396	0.5374	0.0882	0.5374
Dog 4 K9 Duo-Flex (leash)	0.1615	0.1723	0.0332	0.7673	0.4770	0.6462	0.9436	0.0013	0.0922	0.0874	0.0922
Dog 4 Fressnapf	0.0114	0.0044	0.0517	0.0221	0.1442	0.0044	0.0391	0.0005	0.4217	0.0075	0.4217
Dog 4 Fressnapf (leash)	0.3726	0.2348	0.1837	0.3497	0.7784	0.2662	0.6114	0.0383	0.2030	0.4668	0.2030

p>0.05	p≤0.05

Anderson-Darling normality test results

Case	BR Foot Dist	BL Step Distance	BL Step Height	BL Swing Time	BL Swing Ratio	BL Stance Time	BL Stance Ratio	BL Foot Dist	FR Step Distance	FR Step Height	FR Swing Time
Dog 1 reference	0.3739	0.0007	0.0005	0.0019	0.1258	0.0280	0.1258	0.0084	0.6719	0.0046	0.1012
Dog 1 K9 power	0.7626	0.6450	0.0005	0.1158	0.2447	0.0745	0.2447	0.6342	0.5617	0.1342	0.0186
Dog 1 K9 power (leash)	0.4342	0.3132	0.3136	0.0005	0.1160	0.0218	0.1160	0.7063	0.2039	0.7901	0.3402
Dog 1 K9 IDC	0.1782	0.1480	0.6313	0.0068	0.0230	0.0169	0.0230	0.7964	0.1374	0.0901	0.0876
Dog 1 K9 IDC (leash)	0.5052	0.2347	0.0983	0.0666	0.5770	0.3097	0.5770	0.3907	0.2112	0.6201	0.0005
Dog 1 K9 Duo-Flex	0.6214	0.2163	0.0016	0.0909	0.2926	0.2446	0.2926	0.3025	0.0959	0.0005	0.0005
Dog 1 K9 Duo-Flex (leash)	0.5658	0.2570	0.2719	0.0038	0.1277	0.5934	0.1277	0.9570	0.8704	0.0006	0.0005
Dog 2 reference	0.4740	0.0987	0.3187	0.0052	0.3827	0.0437	0.3827	0.2158	0.5331	0.0005	0.0005
Dog 2 K9 power	0.1078	0.4573	0.4114	0.0005	0.0769	0.0014	0.0769	0.6057	0.6830	0.3470	0.0005
Dog 2 K9 power (leash)	0.5621	0.7419	0.9196	0.0094	0.0646	0.0260	0.0646	0.7746	0.3864	0.0047	0.0005
Dog 2 K9 IDC	0.0155	0.1226	0.0974	0.0230	0.6365	0.0244	0.6365	0.1033	0.1980	0.2477	0.0005
Dog 2 K9 IDC (leash)	0.4868	0.2559	0.4262	0.0556	0.5769	0.7150	0.5769	0.6385	0.2662	0.6405	0.0005
Dog 2 K9 Duo-Flex	0.6731	0.7094	0.4112	0.0008	0.3460	0.2257	0.3460	0.3596	0.3644	0.1575	0.0005
Dog 2 K9 Duo-Flex (leash)	0.7992	0.4076	0.2683	0.0483	0.6003	0.8614	0.6003	0.8868	0.8110	0.4649	0.0005
Dog 3 reference	0.4219	0.2876	0.2312	0.0855	0.3339	0.0390	0.3339	0.0143	0.7303	0.0194	0.0005
Dog 3 K9 power	0.0338	0.1122	0.0015	0.0208	0.2872	0.3793	0.2872	0.7093	0.0956	0.1186	0.0091
Dog 3 K9 power (leash)	0.8268	0.9714	0.5238	0.1540	0.3451	0.2891	0.3451	0.4989	0.6855	0.9245	0.0005
Dog 3 K9 IDC	0.4790	0.9002	0.4460	0.0448	0.4208	0.8793	0.4208	0.8067	0.1059	0.2233	0.0015
Dog 3 K9 IDC (leash)	0.0457	0.8178	0.9042	0.1673	0.9579	0.0925	0.9579	0.0295	0.0974	0.0055	0.0082
Dog 4 reference	0.0005	0.0005	0.7251	0.0005	0.0177	0.0005	0.0177	0.0005	0.0079	0.0005	0.0005
Dog 4 K9 power	0.9900	0.5643	0.8319	0.0050	0.6727	0.4196	0.6727	0.5410	0.4322	0.3544	0.5594
Dog 4 K9 power (leash)	0.0191	0.2761	0.6492	0.0101	0.6274	0.0304	0.6274	0.6452	0.2383	0.8220	0.1774
Dog 4 K9 IDC	0.1205	0.5961	0.1465	0.0065	0.3923	0.0747	0.3923	0.8464	0.0452	0.0025	0.0337
Dog 4 K9 IDC (leash)	0.1586	0.2487	0.3713	0.1575	0.9120	0.4797	0.9120	0.5777	0.6677	0.5062	0.4607
Dog 4 K9 Duo-Flex	0.5488	0.0985	0.6271	0.1425	0.0061	0.0064	0.0061	0.0719	0.1401	0.0630	0.0025
Dog 4 K9 Duo-Flex (leash)	0.8669	0.6476	0.3323	0.0018	0.3134	0.1292	0.3134	0.4023	0.4909	0.0724	0.2561
Dog 4 Fressnapf	0.3466	0.9301	0.0014	0.0005	0.0704	0.0020	0.0704	0.0029	0.3876	0.0005	0.0005
Dog 4 Fressnapf (leash)	0.0022	0.1208	0.5744	0.3936	0.6148	0.8202	0.6148	0.1934	0.0116	0.2396	0.0005

p>0.05 p≤0.05

Anderson-Darling normality test results

Case	FR Swing Ratio	FR Stance Time	FR Stance Ratio	FR Foot Dist	FL Step Distance	FL Step Height	FL Swing Time	FL Swing Ratio	FL Stance Time	FL Stance Ratio	FL Foot Dist
Dog 1 reference	0.0009	0.8576	0.0009	0.1809	0.0402	0.1641	0.0005	0.3603	0.4092	0.3603	0.0805
Dog 1 K9 power	0.6529	0.0339	0.6529	0.1433	0.3857	0.0227	0.0052	0.1732	0.2347	0.1732	0.0119
Dog 1 K9 power (leash)	0.2702	0.9435	0.2702	0.5633	0.8056	0.7076	0.0303	0.5580	0.0039	0.5580	0.1913
Dog 1 K9 IDC	0.2736	0.3787	0.2736	0.0449	0.3387	0.0043	0.0708	0.5083	0.0611	0.5083	0.9624
Dog 1 K9 IDC (leash)	0.0005	0.0016	0.0005	0.9126	0.9026	0.0468	0.0528	0.2615	0.0376	0.2615	0.1838
Dog 1 K9 Duo-Flex	0.0005	0.0005	0.0005	0.2243	0.6726	0.0015	0.0181	0.3431	0.4747	0.3431	0.6614
Dog 1 K9 Duo-Flex (leash)	0.0005	0.0005	0.0005	0.1434	0.4516	0.0767	0.0344	0.0033	0.2665	0.0033	0.9360
Dog 2 reference	0.0005	0.0005	0.0005	0.3293	0.1589	0.2181	0.0005	0.0334	0.0200	0.0334	0.7274
Dog 2 K9 power	0.0005	0.0005	0.0005	0.8125	0.2797	0.0310	0.0005	0.2132	0.0063	0.2132	0.4584
Dog 2 K9 power (leash)	0.0005	0.0005	0.0005	0.4841	0.2437	0.0961	0.0019	0.4232	0.0480	0.4232	0.0382
Dog 2 K9 IDC	0.0005	0.0005	0.0005	0.0968	0.8266	0.0112	0.0248	0.0697	0.0012	0.0697	0.2315
Dog 2 K9 IDC (leash)	0.0005	0.0005	0.0005	0.3158	0.3428	0.3590	0.1472	0.2089	0.6811	0.2089	0.0410
Dog 2 K9 Duo-Flex	0.0005	0.0005	0.0005	0.2429	0.3600	0.8176	0.0721	0.1579	0.7355	0.1579	0.3276
Dog 2 K9 Duo-Flex (leash)	0.0005	0.0005	0.0005	0.0720	0.1370	0.6663	0.0005	0.4332	0.4320	0.4332	0.0271
Dog 3 reference	0.1734	0.3977	0.1734	0.0190	0.1898	0.6421	0.0005	0.0722	0.4229	0.0722	0.7058
Dog 3 K9 power	0.0687	0.4727	0.0687	0.0177	0.7898	0.8749	0.0005	0.0934	0.4747	0.0934	0.9433
Dog 3 K9 power (leash)	0.3580	0.9741	0.3580	0.9012	0.8593	0.8154	0.0024	0.6647	0.4645	0.6647	0.8188
Dog 3 K9 IDC	0.1183	0.2298	0.1183	0.0711	0.7758	0.9462	0.0120	0.5936	0.3478	0.5936	0.9411
Dog 3 K9 IDC (leash)	0.4890	0.3393	0.4890	0.8914	0.5068	0.0005	0.0010	0.3104	0.0959	0.3104	0.6975
Dog 4 reference	0.0129	0.0005	0.0129	0.0005	0.0005	0.0005	0.0005	0.0735	0.0005	0.0735	0.0005
Dog 4 K9 power	0.3255	0.3630	0.3255	0.5221	0.8596	0.1612	0.0065	0.5424	0.3494	0.5424	0.4597
Dog 4 K9 power (leash)	0.2663	0.0448	0.2663	0.5744	0.3585	0.4228	0.0810	0.1336	0.0790	0.1336	0.6025
Dog 4 K9 IDC	0.1532	0.6891	0.1532	0.0416	0.6137	0.0005	0.0455	0.2505	0.2149	0.2505	0.1359
Dog 4 K9 IDC (leash)	0.2206	0.6865	0.2206	0.2109	0.9000	0.3331	0.0674	0.7591	0.0138	0.7591	0.7749
Dog 4 K9 Duo-Flex	0.0401	0.0049	0.0401	0.7332	0.0087	0.0428	0.0087	0.6018	0.1032	0.6018	0.1971
Dog 4 K9 Duo-Flex (leash)	0.7752	0.5106	0.7752	0.3931	0.8525	0.4445	0.0005	0.1839	0.4367	0.1839	0.1146
Dog 4 Fressnapf	0.0005	0.0005	0.0005	0.0181	0.2505	0.0005	0.0008	0.2196	0.0920	0.2196	0.4625
Dog 4 Fressnapf (leash)	0.0005	0.0005	0.0005	0.8160	0.5887	0.5113	0.5247	0.2264	0.0402	0.2264	0.0150

$p>0.05$ $p\leq 0.05$

Anderson-Darling normality test results

Case	Back Walking Base	Front Walking Base	T1 hor. ROM	T13 hor. ROM	L7 hor. ROM	T1 sag. ROM	T13 sag. ROM	L7 sag. ROM	FR shoulder ROM	FL shoulder ROM	FR elbow ROM
Dog 1 reference	0.3110	0.8137	0.0005	0.0005	0.7085	0.0005	0.0662	0.0014	0.0042	0.1939	0.2823
Dog 1 K9 power	0.2968	0.7631	0.0005	0.1616	0.3746	0.0077	0.4267	0.0238	0.5128	0.2059	0.5951
Dog 1 K9 power (leash)	0.1217	0.8882	0.0833	0.2294	0.3048	0.0015	0.2718	0.8186	0.2979	0.6483	0.3922
Dog 1 K9 IDC	0.5292	0.6792	0.0039	0.0691	0.5403	0.7992	0.2193	0.8671	0.7754	0.2468	0.7868
Dog 1 K9 IDC (leash)	0.0307	0.9616	0.0159	0.2395	0.5704	0.6171	0.4182	0.9580	0.8297	0.7785	0.8250
Dog 1 K9 Duo-Flex	0.8989	0.9900	0.0619	0.1232	0.3050	0.1636	0.0076	0.1439	0.0189	0.2926	0.7650
Dog 1 K9 Duo-Flex (leash)	0.9668	0.0966	0.0005	0.2108	0.3187	0.0005	0.8020	0.7507	0.0056	0.1769	0.0358
Dog 2 reference	0.4883	0.9778	0.0596	0.2871	0.6633	0.0301	0.0720	0.0967	0.1205	0.7748	0.8882
Dog 2 K9 power	0.8904	0.6273	0.0225	0.1588	0.0407	0.0005	0.4539	0.6614	0.1809	0.4251	0.7806
Dog 2 K9 power (leash)	0.3446	0.0426	0.0871	0.1298		0.0063	0.0461		0.1896	0.2055	0.9671
Dog 2 K9 IDC	0.0127	0.1022	0.2354	0.2495	0.5493	0.0051	0.2728	0.4841	0.3641	0.7356	0.7480
Dog 2 K9 IDC (leash)	0.8637	0.1771	0.1083	0.2467	0.8045	0.0765	0.8069	0.7981	0.0482	0.0517	0.9147
Dog 2 K9 Duo-Flex	0.9277	0.9318	0.0010	0.3874	0.4303	0.0478	0.0215	0.9695	0.5038	0.9260	0.6879
Dog 2 K9 Duo-Flex (leash)	0.9568	0.4025	0.0041	0.0852	0.4977	0.0493	0.0322	0.6014	0.8121	0.6578	0.8965
Dog 3 reference	0.5831	0.0933	0.9064	0.0332	0.5297	0.0005	0.3361	0.0415	0.2551	0.3327	0.4730
Dog 3 K9 power	0.1022	0.6196	0.7205	0.2678	0.0166	0.0005	0.3712	0.2968	0.3588	0.1465	0.3902
Dog 3 K9 power (leash)	0.5322	0.8591	0.2034	0.5949		0.0536	0.8553		0.2746	0.9482	0.6907
Dog 3 K9 IDC	0.5466	0.7553	0.8387	0.9690	0.4071	0.0014	0.3431	0.2953	0.6969	0.1821	0.9819
Dog 3 K9 IDC (leash)	0.2342	0.9002	0.6467	0.6190	0.0309	0.0654	0.1341	0.0746	0.3304	0.0019	0.6386
Dog 4 reference	0.1106	0.0379	0.6854	0.8884	0.0668	0.0005	0.1488	0.0511	0.9376	0.6756	0.0014
Dog 4 K9 power	0.7937	0.4264	0.3193	0.2664	0.3592	0.2314	0.5087	0.2634	0.6995	0.9316	0.2930
Dog 4 K9 power (leash)	0.6831	0.3154	0.8920	0.5043	0.1454	0.3588	0.2967	0.9900	0.9639	0.4879	0.0322
Dog 4 K9 IDC	0.4024	0.1521	0.3145	0.5868	0.1825	0.0859	0.8050	0.0029	0.2755	0.3557	0.6199
Dog 4 K9 IDC (leash)	0.6235	0.5122	0.0436	0.8707	0.0193	0.0204	0.3980	0.5689	0.0398	0.6904	0.5038
Dog 4 K9 Duo-Flex	0.2254	0.6344	0.1857	0.5914	0.8376	0.1779	0.0699	0.7259	0.0093	0.0412	0.3280
Dog 4 K9 Duo-Flex (leash)	0.2458	0.2813	0.1517	0.0032	0.1694	0.3214	0.3477	0.6034	0.8138	0.7248	0.3923
Dog 4 Fressnapf	0.8378	0.8359	0.0024	0.0539	0.7403	0.0005	0.0005	0.0005	0.0005	0.9590	0.0037
Dog 4 Fressnapf (leash)	0.9692	0.5497	0.2916	0.3901	0.0726	0.1151	0.8317	0.4476	0.0522	0.8198	0.1068

$p>0.05$	$p\leq0.05$

Anderson-Darling normality test results

Case	FL elbow ROM	FR carpal ROM	FL carpal ROM	BR hip ROM	BL hip ROM	BR stifle ROM	BL stifle ROM	BR hock ROM	BL hock ROM
Dog 1 reference	0.4064	0.4218	0.0705	0.7614	0.2232	0.9484	0.0005	0.4705	0.0011
Dog 1 K9 power	0.1541	0.3918	0.8982	0.0768	0.6623	0.0049	0.0024	0.0034	0.1378
Dog 1 K9 power (leash)	0.8268	0.1480	0.6005	0.5591	0.2057	0.2580	0.0683	0.9401	0.0262
Dog 1 K9 IDC	0.1938	0.8086	0.1512	0.2642	0.3478	0.6057	0.3954	0.8522	0.2062
Dog 1 K9 IDC (leash)	0.1893	0.0262	0.8540	0.7212	0.3446	0.1776	0.1150	0.0137	0.1230
Dog 1 K9 Duo-Flex	0.0005	0.1911	0.1686	0.3208	0.0187	0.1205	0.3242	0.1330	0.3712
Dog 1 K9 Duo-Flex (leash)	0.3630	0.9362	0.5059	0.5484	0.5613	0.2868	0.0895	0.3641	0.2404
Dog 2 reference	0.1574	0.4708	0.3044	0.7715	0.2215	0.0341	0.0532	0.1829	0.3097
Dog 2 K9 power	0.1273	0.0034	0.7441	0.1125	0.1809	0.1082	0.0059	0.1002	0.0291
Dog 2 K9 power (leash)	0.3542	0.6585	0.2026	0.1845	0.9469	0.5254	0.9184	0.1605	0.4341
Dog 2 K9 IDC	0.8137	0.0173	0.0016	0.0844	0.4169	0.4011	0.0910	0.5719	0.6080
Dog 2 K9 IDC (leash)	0.0024	0.8167	0.2792	0.0321	0.3050	0.1321	0.7140	0.9900	0.7143
Dog 2 K9 Duo-Flex	0.0992	0.9900	0.8132	0.0785	0.7295	0.0647	0.5900	0.3826	0.0349
Dog 2 K9 Duo-Flex (leash)	0.4824	0.9035	0.8046	0.6133	0.1082	0.0607	0.0704	0.7448	0.0681
Dog 3 reference	0.8822	0.0005	0.3665	0.1119	0.0981	0.2477	0.6887	0.3738	0.3712
Dog 3 K9 power	0.8249	0.0005	0.4962	0.6235	0.4667	0.4597	0.6820	0.9623	0.1571
Dog 3 K9 power (leash)	0.1513	0.3684	0.2698	0.1384	0.5785	0.8266	0.6989	0.9545	0.0642
Dog 3 K9 IDC	0.8181	0.0005	0.5457	0.3772	0.3386	0.2085	0.6227	0.2316	0.2424
Dog 3 K9 IDC (leash)	0.1667	0.5578	0.6505	0.0043	0.7954	0.0828	0.1651	0.0005	0.0265
Dog 4 reference	0.0005	0.1237	0.0233	0.6474	0.1267	0.0005	0.0206	0.0633	0.4665
Dog 4 K9 power	0.4601	0.0867	0.7860	0.1805	0.7992	0.1492	0.4222	0.9770	0.5241
Dog 4 K9 power (leash)	0.3317	0.9121	0.3257	0.8850	0.8562	0.8439	0.5740	0.2631	0.1672
Dog 4 K9 IDC	0.1879	0.3237	0.8247	0.0246	0.4998	0.4278	0.1507	0.9446	0.6286
Dog 4 K9 IDC (leash)	0.4408	0.7148	0.5568	0.0035	0.4393	0.1341	0.4824	0.8595	0.8524
Dog 4 K9 Duo-Flex	0.0844	0.4582	0.0204	0.2860	0.0991	0.4998	0.6818	0.9628	0.8612
Dog 4 K9 Duo-Flex (leash)	0.0740	0.6290	0.2258	0.0763	0.7576	0.7337	0.3225	0.6929	0.0692
Dog 4 Fressnapf	0.0969	0.5945	0.9323	0.4111	0.3388	0.0467	0.0674	0.6386	0.9900
Dog 4 Fressnapf (leash)	0.1546	0.1106	0.0104	0.2959	0.0835	0.9790	0.9164	0.7252	0.4440

p>0.05 p≤0.05
