# Supplementary material for: Development of a detailed canine gait analysis method for evaluating harnesses: A pilot study
Source: PLoS One. 2022 Mar 9;17(3):e0264299. doi: 10.1371/journal.pone.0264299 (PMC8906618; doi:10.1371/journal.pone.0264299)
Supplement: S3 Table — Tabular results of the p-values of the two-sided Kolmogorov-Smirnov test for the 53 calculated scalar parameters comparing reference and harness trials, harness and harness+leash trials and reference and harness+leash trials. (PDF) [file pone.0264299.s003.pdf]

Two-sample Kolmogorov-Smirnov test results

Case	Cycle Time	Cadence	Back Stride Distance	Front Stride Distance	Speed	BR Step Distance	BR Step Height	BR Swing Time	BR Swing Ratio	BR Stance Time	BR Stance Ratio
Dog 1 K9 power: ref-harness	0.7032	0.7032	0.0866	0.0029	0.0564	0.4482	0.4482	0.9980	0.8274	0.9246	0.8274
Dog 1 K9 power: harness-leash	0.0205	0.0205	0.0193	0.0004	0.5041	0.2437	0.0701	0.0001	0.0001	0.9435	0.0001
Dog 1 K9 power: leash-ref	0.0291	0.0291	0.0019	0.0009	0.4223	0.6747	0.0164	0.0000	0.0005	0.8429	0.0005
Dog 1 K9 IDC: ref-harness	0.0000	0.0000	0.0000	0.0000	0.0000	0.0002	0.1269	0.7440	0.0002	0.0000	0.0002
Dog 1 K9 IDC: harness-leash	0.6958	0.6958	0.9543	0.9050	0.5548	0.0282	0.0000	0.0029	0.0005	0.0776	0.0005
Dog 1 K9 IDC: leash-ref	0.0000	0.0000	0.0002	0.0008	0.0001	0.4400	0.0000	0.0040	0.0000	0.0000	0.0000
Dog 1 K9 Duo-Flex: ref-harness	0.0000	0.0000	0.0000	0.0000	0.0000	0.0955	0.0002	0.0411	0.0000	0.0000	0.0000
Dog 1 K9 Duo-Flex: harness-leash	0.0003	0.0003	0.0187	0.0002	0.2793	0.1687	0.0029	0.0010	0.0876	0.3003	0.0876
Dog 1 K9 Duo-Flex: leash-ref	0.1647	0.1647	0.0000	0.0000	0.0000	0.0016	0.0000	0.0000	0.0000	0.0000	0.0000
Dog 2 K9 power: ref-harness	0.0384	0.0384	0.0938	0.2492	0.8243	0.0581	0.0010	0.2903	0.3748	0.0384	0.3748
Dog 2 K9 power: harness-leash	0.8328	0.8328	0.0007	0.0003	0.0107	0.0004	0.0230	0.0034	0.1101	0.6318	0.1101
Dog 2 K9 power: leash-ref	0.4272	0.4272	0.0428	0.0168	0.0107	0.3292	0.9181	0.1140	0.0394	0.1939	0.0394
Dog 2 K9 IDC: ref-harness	0.0001	0.0001	0.0367	0.0412	0.0000	0.4864	0.6483	0.0004	0.3862	0.0030	0.3862
Dog 2 K9 IDC: harness-leash	0.5841	0.5841	0.6461	0.5136	0.1270	0.7880	0.1087	0.0477	0.7487	0.9856	0.7487
Dog 2 K9 IDC: leash-ref	0.0000	0.0000	0.0247	0.0349	0.0000	0.1657	0.1657	0.0002	0.7443	0.0019	0.7443
Dog 2 K9 Duo-Flex: ref-harness	0.0304	0.0304	0.0000	0.0000	0.0000	0.0002	0.0000	0.0079	0.0414	0.0642	0.0414
Dog 2 K9 Duo-Flex: harness-leash	0.0016	0.0016	0.0156	0.0804	0.4216	0.0420	0.0046	0.0000	0.1222	0.0384	0.1222
Dog 2 K9 Duo-Flex: leash-ref	0.0000	0.0000	0.0016	0.0001	0.0000	0.0206	0.2519	0.0001	0.2325	0.0001	0.2325
Dog 3 K9 power: ref-harness	0.7557	0.7557	0.1446	0.1662	0.9980	0.7472	0.0003	0.9734	0.2712	0.5542	0.2712
Dog 3 K9 power: harness-leash	0.5338	0.5338	0.0007	0.0384	0.8087	0.0083	0.0311	0.4049	0.1446	0.2964	0.1446
Dog 3 K9 power: leash-ref	0.4787	0.4787	0.0266	0.0416	0.6498	0.0225	0.7821	0.6759	0.3721	0.6136	0.3721
Dog 3 K9 IDC: ref-harness	0.4237	0.4237	0.8681	0.7636	0.7931	0.9225	0.0194	0.9889	0.1154	0.5696	0.1154
Dog 3 K9 IDC: harness-leash	0.1810	0.1810	0.0189	0.0034	0.8158	0.0019	0.3952	0.0704	0.0016	0.0435	0.0016
Dog 3 K9 IDC: leash-ref	0.2463	0.2463	0.0001	0.0426	0.3825	0.0001	0.5713	0.0466	0.0303	0.1872	0.0303
Dog 4 K9 power: ref-harness	0.0000	0.0000	0.0000	0.0000	0.8257	0.0000	0.0000	0.0000	0.0007	0.0000	0.0007
Dog 4 K9 power: harness-leash	0.0000	0.0000	0.0000	0.0000	0.8281	0.0001	0.1106	0.0000	0.5407	0.0000	0.5407
Dog 4 K9 power: leash-ref	0.0000	0.0000	0.0009	0.0006	0.8334	0.0003	0.0000	0.0000	0.0009	0.0041	0.0009
Dog 4 K9 IDC: ref-harness	0.0000	0.0000	0.0000	0.0000	0.4245	0.0000	0.0000	0.0000	0.0065	0.0000	0.0065
Dog 4 K9 IDC: harness-leash	0.0002	0.0002	0.0000	0.0000	0.5088	0.0197	0.4808	0.0173	0.0287	0.0000	0.0287
Dog 4 K9 IDC: leash-ref	0.0000	0.0000	0.0000	0.0002	0.8362	0.0000	0.0000	0.0000	0.0000	0.0141	0.0000
Dog 4 K9 Duo-Flex: ref-harness	0.0000	0.0000	0.0000	0.0000	0.3296	0.0000	0.8676	0.0000	0.0077	0.0000	0.0077
Dog 4 K9 Duo-Flex: harness-leash	0.1746	0.1746	0.5628	0.7974	0.5309	0.5628	0.0138	0.3320	0.5628	0.8210	0.5628
Dog 4 K9 Duo-Flex: leash-ref	0.0000	0.0000	0.0000	0.0000	0.9537	0.0000	0.0018	0.0000	0.0150	0.0000	0.0150
Dog 4 Fressnapf: ref-harness	0.0000	0.0000	0.0001	0.0000	0.7473	0.0323	0.0000	0.0000	0.0017	0.0002	0.0017
Dog 4 Fressnapf: harness-leash	0.0009	0.0009	0.0002	0.0257	0.8900	0.2139	0.6025	0.0037	0.1588	0.1460	0.1588
Dog 4 Fressnapf: leash-ref	0.0000	0.0000	0.0000	0.0000	0.7410	0.0547	0.0000	0.0000	0.0072	0.0000	0.0072

$p>0.05$ $p\leq 0.05$

Two-sample Kolmogorov-Smirnov test results

Case	BR Foot Dist	BL Step Distance	BL Step Height	BL Swing Time	BL Swing Ratio	BL Stance Time	BL Stance Ratio	BL Foot Dist	FR Step Distance	FR Step Height	FR Swing Time
Dog 1 K9 power: ref-harness	0.5719	0.7032	0.1806	0.9246	0.8274	0.4482	0.8274	0.7032	0.2741	0.9246	0.3403
Dog 1 K9 power: harness-leash	0.0057	0.0001	0.1738	0.0007	0.1815	0.1663	0.1815	0.0006	0.7936	0.0666	0.7809
Dog 1 K9 power: leash-ref	0.0054	0.0001	0.0823	0.0000	0.0444	0.5163	0.0444	0.0009	0.5004	0.1245	0.6747
Dog 1 K9 IDC: ref-harness	0.0018	0.0596	0.0022	0.3755	0.0000	0.0000	0.0000	0.0004	0.0001	0.0256	0.0000
Dog 1 K9 IDC: harness-leash	0.0002	0.0051	0.0004	0.0017	0.0007	0.0776	0.0007	0.0001	0.3834	0.0142	0.0235
Dog 1 K9 IDC: leash-ref	0.0087	0.0000	0.0000	0.0002	0.0000	0.0000	0.0000	0.0012	0.0054	0.0867	0.0293
Dog 1 K9 Duo-Flex: ref-harness	0.4139	0.0000	0.0000	0.7256	0.0000	0.0000	0.0000	0.0005	0.0014	0.0000	0.0000
Dog 1 K9 Duo-Flex: harness-leash	0.1687	0.0876	0.0876	0.0003	0.4903	0.0420	0.4903	0.0010	0.4656	0.0010	0.0000
Dog 1 K9 Duo-Flex: leash-ref	0.2046	0.0000	0.0011	0.0000	0.0000	0.0001	0.0000	0.4711	0.0000	0.1816	0.1312
Dog 2 K9 power: ref-harness	0.0442	0.8953	0.7332	0.0122	0.7391	0.0559	0.7391	0.0270	0.1160	0.0007	0.3605
Dog 2 K9 power: harness-leash	0.9852	0.7016	0.2739	0.0000	0.0000	0.0372	0.0000	0.0003	0.0004	0.0004	0.2739
Dog 2 K9 power: leash-ref	0.4272	0.6614	0.2195	0.0000	0.0000	0.0115	0.0000	0.0002	0.0168	0.0000	0.0167
Dog 2 K9 IDC: ref-harness	0.1383	0.0557	0.0187	0.1116	0.0179	0.0008	0.0179	0.0736	0.0045	0.0000	0.0004
Dog 2 K9 IDC: harness-leash	0.1372	0.8069	0.0004	0.0009	0.0110	0.8069	0.0110	0.0009	0.1952	0.0853	0.5637
Dog 2 K9 IDC: leash-ref	0.1657	0.0784	0.1931	0.0000	0.4588	0.0093	0.4588	0.0006	0.4181	0.0336	0.0003
Dog 2 K9 Duo-Flex: ref-harness	0.0072	0.0159	0.0007	0.0013	0.0028	0.0173	0.0028	0.0002	0.0002	0.0002	0.0000
Dog 2 K9 Duo-Flex: harness-leash	0.1090	0.7063	0.8088	0.0000	0.0014	0.6795	0.0014	0.9216	0.0003	0.0861	0.0164
Dog 2 K9 Duo-Flex: leash-ref	0.2724	0.0302	0.0016	0.0000	0.7149	0.0112	0.7149	0.0000	0.8738	0.2586	0.0000
Dog 3 K9 power: ref-harness	0.0829	0.0829	0.0001	0.7213	0.8582	0.3657	0.8582	0.0192	0.1159	0.0001	0.9643
Dog 3 K9 power: harness-leash	0.0007	0.0966	0.0083	0.6038	0.0083	0.1750	0.0083	0.0047	0.0762	0.2506	0.4049
Dog 3 K9 power: leash-ref	0.0021	0.8867	0.9360	0.2523	0.0013	0.2818	0.0013	0.0034	0.1139	0.0202	0.3721
Dog 3 K9 IDC: ref-harness	0.2276	0.8830	0.0682	0.7646	0.7832	0.7265	0.7832	0.1289	0.1599	0.0469	0.1683
Dog 3 K9 IDC: harness-leash	0.0000	0.9620	0.0021	0.0435	0.0169	0.0481	0.0169	0.0000	0.2445	0.1810	0.0772
Dog 3 K9 IDC: leash-ref	0.0000	0.1785	0.1139	0.0040	0.0010	0.0112	0.0010	0.0000	0.0002	0.0002	0.2574
Dog 4 K9 power: ref-harness	0.0000	0.0000	0.0000	0.0000	0.2313	0.0000	0.2313	0.0000	0.0000	0.0000	0.0000
Dog 4 K9 power: harness-leash	0.0000	0.0000	0.8622	0.0001	0.0006	0.0000	0.0006	0.0000	0.0001	0.0116	0.0000
Dog 4 K9 power: leash-ref	0.0310	0.0897	0.0003	0.0000	0.0000	0.2313	0.0000	0.0075	0.0089	0.0002	0.0111
Dog 4 K9 IDC: ref-harness	0.0000	0.0000	0.0003	0.0000	0.2542	0.0000	0.2542	0.0000	0.0000	0.0000	0.0000
Dog 4 K9 IDC: harness-leash	0.0000	0.0000	0.2179	0.0731	0.0345	0.0000	0.0345	0.0000	0.0122	0.0000	0.0066
Dog 4 K9 IDC: leash-ref	0.5097	0.0059	0.0000	0.0000	0.0010	0.0049	0.0010	0.4738	0.5136	0.0591	0.0020
Dog 4 K9 Duo-Flex: ref-harness	0.0000	0.0013	0.2226	0.0003	0.0742	0.0000	0.0742	0.0000	0.0000	0.2366	0.0006
Dog 4 K9 Duo-Flex: harness-leash	0.1746	0.5628	0.0000	0.0004	0.0000	0.3320	0.0000	0.0001	0.0108	0.0015	0.0828
Dog 4 K9 Duo-Flex: leash-ref	0.0000	0.0000	0.0002	0.0000	0.0000	0.0000	0.0000	0.1565	0.0000	0.0000	0.0000
Dog 4 Fressnapf: ref-harness	0.0088	0.0001	0.0000	0.0000	0.0009	0.0053	0.0009	0.0062	0.0338	0.0000	0.0018
Dog 4 Fressnapf: harness-leash	0.1566	0.1174	0.0084	0.0000	0.0066	0.1588	0.0066	0.1798	0.6145	0.0010	0.0053
Dog 4 Fressnapf: leash-ref	0.0038	0.0002	0.0000	0.0000	0.0030	0.0145	0.0030	0.8346	0.0456	0.0000	0.0000

$p>0.05$ $p\leq 0.05$

Two-sample Kolmogorov-Smirnov test results

Case	FR Swing Ratio	FR Stance Time	FR Stance Ratio	FR Foot Dist	FL Step Distance	FL Step Height	FL Swing Time	FL Swing Ratio	FL Stance Time	FL Stance Ratio	FL Foot Dist
Dog 1 K9 power: ref-harness	0.0578	0.5719	0.0578	0.8274	0.0148	0.0376	0.4482	0.1266	0.9246	0.1266	0.5719
Dog 1 K9 power: harness-leash	0.0347	0.5177	0.0347	0.7964	0.0000	0.0065	0.8678	0.3327	0.0110	0.3327	0.0007
Dog 1 K9 power: leash-ref	0.0011	0.7794	0.0011	0.7279	0.0000	0.0000	0.4666	0.4994	0.0054	0.4994	0.0012
Dog 1 K9 IDC: ref-harness	0.0000	0.0000	0.0000	0.0004	0.0000	0.6764	0.0283	0.0000	0.0000	0.0000	0.0223
Dog 1 K9 IDC: harness-leash	0.0562	0.0117	0.0562	0.1003	0.5247	0.0005	0.0358	0.0063	0.5581	0.0063	0.7129
Dog 1 K9 IDC: leash-ref	0.0096	0.0401	0.0096	0.1919	0.0004	0.0000	0.0461	0.0093	0.0002	0.0093	0.0487
Dog 1 K9 Duo-Flex: ref-harness	0.0005	0.0000	0.0005	0.0031	0.0000	0.0075	0.9961	0.0000	0.0000	0.0000	0.0000
Dog 1 K9 Duo-Flex: harness-leash	0.7198	0.0003	0.7198	0.9168	0.0029	0.0000	0.7198	0.0029	0.0010	0.0029	0.0077
Dog 1 K9 Duo-Flex: leash-ref	0.0010	0.2246	0.0010	0.0035	0.0000	0.0000	0.6887	0.0663	0.1567	0.0663	0.0000
Dog 2 K9 power: ref-harness	0.0075	0.0107	0.0075	0.0442	0.0858	0.1513	0.0827	0.0000	0.0080	0.0000	0.0060
Dog 2 K9 power: harness-leash	0.0728	0.8881	0.0728	0.4957	0.0178	0.0001	0.1944	0.6318	0.6318	0.6318	0.1101
Dog 2 K9 power: leash-ref	0.0167	0.0200	0.0167	0.1497	0.3115	0.0428	0.0096	0.0464	0.0363	0.0464	0.0004
Dog 2 K9 IDC: ref-harness	0.1589	0.1589	0.1589	0.1383	0.2582	0.0367	0.0000	0.5224	0.0001	0.5224	0.0557
Dog 2 K9 IDC: harness-leash	0.3149	0.3149	0.3149	0.3298	0.0068	0.3298	0.9685	0.8069	0.4659	0.8069	0.0162
Dog 2 K9 IDC: leash-ref	0.9602	0.0304	0.9602	0.0933	0.0003	0.0933	0.0000	0.6571	0.0015	0.6571	0.0000
Dog 2 K9 Duo-Flex: ref-harness	0.0000	0.0000	0.0000	0.0019	0.0000	0.0031	0.1926	0.1711	0.2554	0.1711	0.0002
Dog 2 K9 Duo-Flex: harness-leash	0.9369	0.0017	0.9369	0.1132	0.9369	0.9216	0.0005	0.2388	0.0479	0.2388	0.0058
Dog 2 K9 Duo-Flex: leash-ref	0.0000	0.0000	0.0000	0.0314	0.0000	0.0051	0.0000	0.4787	0.0002	0.4787	0.0003
Dog 3 K9 power: ref-harness	0.3321	0.3869	0.3321	0.7809	0.0056	0.0297	0.0851	0.6774	0.9676	0.6774	0.5286
Dog 3 K9 power: harness-leash	0.6038	0.4671	0.6038	0.0627	0.0311	0.1750	0.8124	0.9577	0.5338	0.9577	0.0144
Dog 3 K9 power: leash-ref	0.9180	0.9583	0.9180	0.0161	0.4930	0.7375	0.5981	0.1925	0.5076	0.1925	0.0010
Dog 3 K9 IDC: ref-harness	0.8189	0.8971	0.8189	0.8013	0.0295	0.0725	0.0079	0.1963	0.9943	0.1963	0.4942
Dog 3 K9 IDC: harness-leash	0.3034	0.3707	0.3034	0.0012	0.0085	0.0024	0.0028	0.0846	0.5305	0.0846	0.0151
Dog 3 K9 IDC: leash-ref	0.6075	0.6808	0.6075	0.0009	0.6075	0.1139	0.7876	0.9809	0.4347	0.9809	0.0004
Dog 4 K9 power: ref-harness	0.2715	0.0000	0.2715	0.0000	0.0000	0.0000	0.0000	0.0000	0.0000	0.0000	0.0000
Dog 4 K9 power: harness-leash	0.8622	0.0000	0.8622	0.0000	0.0001	0.0389	0.0000	0.1106	0.0000	0.1106	0.0000
Dog 4 K9 power: leash-ref	0.4954	0.0000	0.4954	0.6914	0.0012	0.0000	0.9465	0.0013	0.0000	0.0013	0.0000
Dog 4 K9 IDC: ref-harness	0.0010	0.0000	0.0010	0.0000	0.0000	0.0000	0.0000	0.0000	0.0000	0.0000	0.0000
Dog 4 K9 IDC: harness-leash	0.0731	0.0002	0.0731	0.0042	0.0001	0.0006	0.0053	0.5142	0.0001	0.5142	0.0000
Dog 4 K9 IDC: leash-ref	0.0751	0.0000	0.0751	0.0012	0.0000	0.0000	0.9028	0.0000	0.0000	0.0000	0.0000
Dog 4 K9 Duo-Flex: ref-harness	0.0189	0.0000	0.0189	0.0000	0.0002	0.0000	0.2158	0.0000	0.0000	0.0000	0.0000
Dog 4 K9 Duo-Flex: harness-leash	0.3320	0.0828	0.3320	0.1746	0.1746	0.3320	0.0049	0.0828	0.8210	0.0828	0.5628
Dog 4 K9 Duo-Flex: leash-ref	0.4242	0.0000	0.4242	0.0000	0.0015	0.0000	0.0000	0.0006	0.0000	0.0006	0.0000
Dog 4 Fressnapf: ref-harness	0.0301	0.0000	0.0301	0.0000	0.0000	0.0000	0.0721	0.0010	0.0000	0.0010	0.0000
Dog 4 Fressnapf: harness-leash	0.0951	0.0027	0.0951	0.0379	0.0053	0.3822	0.2253	0.8684	0.0936	0.8684	0.5921
Dog 4 Fressnapf: leash-ref	0.1087	0.0000	0.1087	0.0000	0.0000	0.0000	0.0778	0.0547	0.0000	0.0547	0.0003

$p>0.05$ $p\leq 0.05$

Two-sample Kolmogorov-Smirnov test results

Case	Back Walking Base	Front Walking Base	T1 hor. ROM	T13 hor. ROM	L7 hor. ROM	T1 sag. ROM	T13 sag. ROM	L7 sag. ROM	FR shoulder ROM	FL shoulder ROM	FR elbow ROM
Dog 1 K9 power: ref-harness	0.7032	0.2512	0.4482	0.0000	0.0376	0.3403	0.4482	0.0376	0.0000	0.0000	0.0239
Dog 1 K9 power: harness-leash	0.0193	0.3845	0.6831	0.0000	0.0000	0.0057	0.0996	0.0666	0.0007	0.0028	0.2748
Dog 1 K9 power: leash-ref	0.0372	0.0394	0.9575	0.0000	0.0000	0.0011	0.3474	0.0003	0.0000	0.0000	0.0110
Dog 1 K9 IDC: ref-harness	0.1613	0.3834	0.9640	0.0000	0.9820	0.8422	0.0187	0.9206	0.0000	0.0000	0.7721
Dog 1 K9 IDC: harness-leash	0.0001	0.0425	0.5083	0.0004	0.0172	0.0266	0.1550	0.0002	0.1945	0.3458	0.7129
Dog 1 K9 IDC: leash-ref	0.0004	0.3129	0.3129	0.0000	0.0261	0.0310	0.7087	0.0004	0.0000	0.0000	0.6244
Dog 1 K9 Duo-Flex: ref-harness	0.0033	0.6701	0.9999	0.0000	0.6608	0.4061	0.3756	0.9407	0.0000	0.0000	0.0031
Dog 1 K9 Duo-Flex: harness-leash	0.7198	0.3003	0.1687	0.0000	0.0000	0.0187	0.0077	0.0876	0.0000	0.0000	0.0003
Dog 1 K9 Duo-Flex: leash-ref	0.0000	0.6328	0.1246	0.0000	0.0000	0.0239	0.0000	0.0231	0.0000	0.0000	0.2246
Dog 2 K9 power: ref-harness	0.5124	0.1061	0.0000	0.0000	0.0000	0.0000	0.0000	0.1833	0.1348	0.1197	0.0905
Dog 2 K9 power: harness-leash	0.0000	0.0005	0.0081	0.0000		0.7016	0.0000		0.6318	0.0034	0.0000
Dog 2 K9 power: leash-ref	0.0000	0.0017	0.0079	0.1939		0.0002	0.2945		0.9325	0.0017	0.0000
Dog 2 K9 IDC: ref-harness	0.0270	0.0766	0.0074	0.0000	0.1383	0.0055	0.0399	0.0827	0.0000	0.0000	0.0708
Dog 2 K9 IDC: harness-leash	0.0086	0.0007	0.1909	0.0389	0.0003	0.7487	0.5237	0.0005	0.0034	0.0073	0.2233
Dog 2 K9 IDC: leash-ref	0.0597	0.0450	0.0104	0.0000	0.0000	0.0083	0.0104	0.0041	0.0000	0.0000	0.2238
Dog 2 K9 Duo-Flex: ref-harness	0.0907	0.3491	0.0001	0.0000	0.9038	0.9638	0.0000	0.9638	0.5702	0.0000	0.0001
Dog 2 K9 Duo-Flex: harness-leash	0.0134	0.0001	0.0827	0.0000	0.0000	0.0827	0.0000	0.0000	0.1268	0.0000	0.4344
Dog 2 K9 Duo-Flex: leash-ref	0.2941	0.0015	0.0541	0.0000	0.0000	0.0240	0.0132	0.0000	0.4394	0.0000	0.0000
Dog 3 K9 power: ref-harness	0.3192	0.9097	0.4315	0.0007	0.0463	0.8721	0.5542	0.4089	0.0002	0.0000	0.4708
Dog 3 K9 power: harness-leash	0.4049	0.0966	0.4671	0.0001		0.0311	0.0000		0.0001	0.0002	0.0627
Dog 3 K9 power: leash-ref	0.8506	0.0161	0.6292	0.0000		0.1637	0.0000		0.0000	0.0000	0.0135
Dog 3 K9 IDC: ref-harness	0.5504	0.0770	0.0682	0.0000	0.0181	0.8189	0.5127	0.6086	0.0004	0.0000	0.4760
Dog 3 K9 IDC: harness-leash	0.0007	0.0290	0.0004	0.1542	0.0000	0.8241	0.0234	0.2830	0.0000	0.2452	0.0001
Dog 3 K9 IDC: leash-ref	0.0000	0.0001	0.0011	0.0002	0.0000	0.3737	0.0004	0.0873	0.0000	0.0000	0.0006
Dog 4 K9 power: ref-harness	0.6748	0.0000	0.0000	0.0000	0.1440	0.1721	0.1039	0.3048	0.0000	0.0000	0.3048
Dog 4 K9 power: harness-leash	0.0116	0.0000	0.5407	0.2672	0.5407	0.8622	0.0006	0.0116	0.2672	0.5407	0.5407
Dog 4 K9 power: leash-ref	0.0016	0.0002	0.0000	0.0000	0.2715	0.1089	0.0000	0.0172	0.0000	0.0000	0.3165
Dog 4 K9 IDC: ref-harness	0.0036	0.0000	0.0000	0.0000	0.0320	0.0513	0.2542	0.2542	0.0000	0.0000	0.0114
Dog 4 K9 IDC: harness-leash	0.7777	0.0066	0.0082	0.0731	0.0345	0.0859	0.9490	0.3192	0.0124	0.1006	0.3877
Dog 4 K9 IDC: leash-ref	0.0016	0.0020	0.0000	0.0000	0.0008	0.7289	0.3746	0.1002	0.0000	0.0000	0.0016
Dog 4 K9 Duo-Flex: ref-harness	0.9718	0.1150	0.0319	0.3535	0.0000	0.8952	0.6977	0.0464	0.0000	0.0000	0.0060
Dog 4 K9 Duo-Flex: harness-leash	0.0000	0.1746	0.0000	0.3320	0.3320	0.0828	0.0000	0.3320	0.0000	0.0000	0.9786
Dog 4 K9 Duo-Flex: leash-ref	0.0000	0.0001	0.0000	0.0347	0.0000	0.1191	0.0000	0.0011	0.0000	0.0000	0.0003
Dog 4 Fressnapf: ref-harness	0.2178	0.0000	0.0000	0.0000	0.0000	0.0002	0.0002	0.0000	0.0000	0.0000	0.0001
Dog 4 Fressnapf: harness-leash	0.0004	0.0110	0.9890	0.0014	0.0399	0.5109	0.0867	0.4722	0.0000	0.0000	0.5663
Dog 4 Fressnapf: leash-ref	0.0002	0.0000	0.0000	0.0000	0.3701	0.0041	0.5995	0.2542	0.0000	0.0004	0.0547

$p>0.05$ $p\leq 0.05$

Two-sample Kolmogorov-Smirnov test results

Case	FL elbow ROM	FR carpal ROM	FL carpal ROM	BR hip ROM	BL hip ROM	BR stifle ROM	BL stifle ROM	BR hock ROM	BL hock ROM
Dog 1 K9 power: ref-harness	0.0000	0.0000	0.4482	0.7032	0.0053	0.1806	0.0866	0.0376	0.0239
Dog 1 K9 power: harness-leash	0.9847	0.0003	0.0065	0.0000	0.0000	0.8116	0.4864	0.0006	0.0001
Dog 1 K9 power: leash-ref	0.0199	0.0000	0.0175	0.0000	0.0000	0.6926	0.0134	0.0000	0.0008
Dog 1 K9 IDC: ref-harness	0.0000	0.1613	0.2829	0.0000	0.0001	0.2640	0.4852	0.4411	0.0032
Dog 1 K9 IDC: harness-leash	0.0000	0.1860	0.0005	0.0000	0.0001	0.0000	0.0117	0.0004	0.0023
Dog 1 K9 IDC: leash-ref	0.5825	0.5251	0.0011	0.0025	0.0046	0.0000	0.0049	0.0001	0.0067
Dog 1 K9 Duo-Flex: ref-harness	0.0000	0.2996	0.0048	0.1454	0.3061	0.2996	0.3467	0.1529	0.0981
Dog 1 K9 Duo-Flex: harness-leash	0.0010	0.4903	0.0876	0.0000	0.0000	0.3003	0.0000	0.0000	0.0000
Dog 1 K9 Duo-Flex: leash-ref	0.9407	0.8887	0.0000	0.0000	0.0000	0.1092	0.0000	0.0000	0.0000
Dog 2 K9 power: ref-harness	0.1721	0.0001	0.0001	0.0000	0.0400	0.0099	0.3605	0.1721	0.0001
Dog 2 K9 power: harness-leash	0.0000	0.0000	0.0000	0.0000	0.0000	0.0000	0.7696	0.0000	0.0000
Dog 2 K9 power: leash-ref	0.0000	0.0000	0.0000	0.0000	0.0000	0.0000	0.7345	0.0001	0.0000
Dog 2 K9 IDC: ref-harness	0.3967	0.0535	0.0082	0.0708	0.0827	0.8427	0.1200	0.0007	0.0007
Dog 2 K9 IDC: harness-leash	0.0446	0.0020	0.0110	0.0000	0.0020	0.0086	0.8598	0.3775	0.0020
Dog 2 K9 IDC: leash-ref	0.0004	0.0000	0.0000	0.0000	0.0450	0.0009	0.3837	0.1657	0.0000
Dog 2 K9 Duo-Flex: ref-harness	0.0000	0.0000	0.0000	0.0000	0.9545	0.0123	0.0847	0.0004	0.0003
Dog 2 K9 Duo-Flex: harness-leash	0.0367	0.0367	0.9973	0.0000	0.0000	0.0000	0.1132	0.0156	0.0000
Dog 2 K9 Duo-Flex: leash-ref	0.0000	0.0000	0.0000	0.0000	0.0000	0.0000	0.0644	0.0000	0.0000
Dog 3 K9 power: ref-harness	0.9706	0.3727	0.3727	0.5202	0.0125	0.0014	0.0198	0.3942	0.1746
Dog 3 K9 power: harness-leash	0.0035	0.2506	0.0627	0.0000	0.0000	0.9577	0.9813	0.0966	0.0188
Dog 3 K9 power: leash-ref	0.0007	0.4369	0.5674	0.0000	0.0000	0.0296	0.0523	0.3248	0.0578
Dog 3 K9 IDC: ref-harness	0.0817	0.3015	0.0276	0.7071	0.8189	0.0469	0.0412	0.1773	0.4069
Dog 3 K9 IDC: harness-leash	0.0075	0.0846	0.0005	0.0000	0.0000	0.5305	0.0014	0.0000	0.0925
Dog 3 K9 IDC: leash-ref	0.1962	0.0058	0.1701	0.0000	0.0000	0.0029	0.0000	0.0009	0.0043
Dog 4 K9 power: ref-harness	0.0000	0.2823	0.2934	0.2043	0.0598	0.0000	0.0000	0.1255	0.0152
Dog 4 K9 power: harness-leash	0.8622	0.0389	0.0030	0.0001	0.0000	0.0000	0.1106	0.0006	0.0000
Dog 4 K9 power: leash-ref	0.0000	0.0044	0.0000	0.0000	0.0000	0.0206	0.0007	0.0000	0.0000
Dog 4 K9 IDC: ref-harness	0.3521	0.0000	0.0000	0.1210	0.0194	0.0000	0.0000	0.0065	0.0799
Dog 4 K9 IDC: harness-leash	0.0413	0.0141	0.0009	0.0000	0.0000	0.0000	0.0018	0.0141	0.0000
Dog 4 K9 IDC: leash-ref	0.0010	0.0005	0.0000	0.0000	0.0000	0.0146	0.4738	0.0181	0.0001
Dog 4 K9 Duo-Flex: ref-harness	0.0000	0.7344	0.0427	0.5377	0.2993	0.0006	0.2092	0.0131	0.1785
Dog 4 K9 Duo-Flex: harness-leash	0.0000	0.0000	0.0000	0.0000	0.0000	0.0015	0.0049	0.1746	0.0000
Dog 4 K9 Duo-Flex: leash-ref	0.0000	0.0000	0.0000	0.0000	0.0000	0.6108	0.0000	0.8047	0.0000
Dog 4 Fressnapf: ref-harness	0.0000	0.0000	0.0051	0.0000	0.0000	0.0000	0.0197	0.0483	0.0018
Dog 4 Fressnapf: harness-leash	0.3298	0.0040	0.0002	0.0003	0.0000	0.0000	0.0789	0.4580	0.0007
Dog 4 Fressnapf: leash-ref	0.0000	0.0000	0.0035	0.0000	0.0000	0.3912	0.0700	0.0356	0.0000

$p>0.05$ $p\leq0.05$
