# Supplementary material for: Development of a detailed canine gait analysis method for evaluating harnesses: A pilot study
Source: PLoS One. 2022 Mar 9;17(3):e0264299. doi: 10.1371/journal.pone.0264299 (PMC8906618; doi:10.1371/journal.pone.0264299)
Supplement: Attachment — Submitted filename: rev2 - response_for_editor_K9.pdf [file pone.0264299.s004.pdf]

RESPONSE TO EDITOR

on the article titled

Development of a detailed canine gait analysis method for evaluating harnesses: a pilot study

submitted to the peer-reviewed journal

PLOS ONE

Dear Dr. Tomaszewska,

We have received your comments of your article, and we would like to thank you for your extraordinarily thorough and relevant suggestions! In the following section we will address each point you raised to the best of our abilities.

Journal Requirements:

1. *Please ensure that your manuscript meets PLOS ONE's style requirements, including those for file naming. The PLOS ONE style templates can be found at* https://journals.plos.org/plosone/s/file?id=wjVg/PLOSOne_formatting_sample_main_body.pdf *and* https://journals.plos.org/plosone/s/file?id=ba62/PLOSOne_formatting_sample_title_authors_affiliations.pdf

   We have used the official Latex template of PLOSE ONE. The file naming and labelling have been revised according to the style templates.

2. *In your Methods section, please provide additional details regarding participant consent from the owners of the animals. In the ethics statement in the Methods and online submission information, please ensure that you have specified (1) whether consent was informed and (2) what type you obtained (for instance, written or verbal). If the need for consent was waived by the ethics committee, please include this information.*

   Thank you for bringing our attention to this point! The ethical permit required for the measurements was approved by the Semmelweis University Regional and Institutional Committee of Science which is a part of the Hungarian National Science and Research Ethics Committee. Moreover, we have tried to clarify what information of the consent was verbally communicated to the participant and what required written consent.

   > All dogs participated in the measurement with their owners, who have given their written consent to participate in the experiment after they were informed verbally about all aspects of it (e.g. hazardous situations, instrumentation, use of media contents, etc.). The study was approved by the National Science and Research Ethics Committee (Hungary) (21/2015).

3. *We note that Figures 1,2,3 and 4 in your submission contain copyrighted images. All PLOS content is published under the Creative Commons Attribution License (CC BY 4.0), which means that the manuscript, images, and Supporting Information files will be freely available online, and any third party is permitted to access, download, copy, distribute, and use these materials in any way, even commercially, with proper attribution. For more information, see our copyright guidelines:* *http://journals.plos.org/plosone/s/licenses-and-copyright*.

We require you to either (1) present written permission from the copyright holder to publish these figures specifically under the CC BY 4.0 license, or (2) remove the figures from your submission:

a. You may seek permission from the original copyright holder of Figures 1,2,3 and 4 to publish the content specifically under the CC BY 4.0 license.

We recommend that you contact the original copyright holder with the Content Permission Form (http://journals.plos.org/plosone/s/file?id=7c09/content-permission-form.pdf) and the following text:

"I request permission for the open-access journal PLOS ONE to publish XXX under the Creative Commons Attribution License (CCAL) CC BY 4.0 (http://creativecommons.org/licenses/by/4.0/). Please be aware that this license allows unrestricted use and distribution, even commercially, by third parties. Please reply and provide explicit written permission to publish XXX under a CC BY license and complete the attached form."

Please upload the completed Content Permission Form or other proof of granted permissions as an "Other" file with your submission.

In the figure caption of the copyrighted figure, please include the following text: "Reprinted from [ref] under a CC BY license, with permission from [name of publisher], original copyright [original copyright year]."

b. If you are unable to obtain permission from the original copyright holder to publish these figures under the CC BY 4.0 license or if the copyright holder's requirements are incompatible with the CC BY 4.0 license, please either i) remove the figure or ii) supply a replacement figure that complies with the CC BY 4.0 license. Please check copyright information on all replacement figures and update the figure caption with source information. If applicable, please specify in the figure caption text when a figure is similar but not identical to the original image and is therefore for illustrative purposes only.

Thank you for pointing out the proper use of the copyright items, we appreciate the detailed instructions.
Figure 1 is an image taken during the measurement by us. The participants agreed, when they signed the consent form, that the photographic material taken during the measurements will be used for documentary and publication purposes. Since there is no distinct brand label on the harness (on Figure 1), the owner of Julius-K9 has also confirmed that the signed consent form is sufficient to print the image, no further permission is required from their side.

We have asked for permission from Julius-K9 to use their images in Figure 2. The figure caption was extended with the above mentioned text, and the signed permission will be uploaded during the revision process.

> **Fig. 1. Studied Julius-K9® harnesses.** a) Julius-K9® Duo-Flex harness; b) Julius-K9® IDC harness; c) Julius-K9® power harness. Note, that the measured harnesses were manufactured without the light-reflexive materials. Reprinted from https://julius-k9.com/en/ under a CC BY license, with permission from Julius-K9®, original copyright (1997-2020)

Unfortunately, our wording in the second paragraph of the 'Measurement setup' subsection made it look like that the used marker placement setup was taken over from Hogy et al. as well as the illustrative figure (Figure 3). However, the figure is an illustrative representation of the marker set, and it was created by ourselves for this manuscript applying our own photo content. The caption of this figure has been reworded and we have added the proper reference.

Fig. 3. Marker-set of 25 reflexive markers. Note, that markers on the left side are not shown. Illustrative purpose only, the marker placement procedure was carried out according to Hogy et al. [22].

Figure 4, like the one following it (Fig. 5.), is a self-made figure, which was made from the recorded measurement data. Therefore, we believe the original copyright belong to us. Since the above description did not make it clear to us, we would like to kindly ask the Editors some more detail about Figure 4, what may be the missing permission or supplementary information that would be required to publish the figure.

Based on your comments we have revised our manuscript, and we hope that our revisions prove to be satisfactory! Thank you again for your insightful critique. It helped us greatly in bringing our research up to par with the standards expected by the scientific community!

Best regards:

Zsófia Pálya, Kristóf Rácz, Gergely Nagymáté & Rita M. Kiss
