# Supplementary material for: Development of a detailed canine gait analysis method for evaluating harnesses: A pilot study
Source: PLoS One. 2022 Mar 9;17(3):e0264299. doi: 10.1371/journal.pone.0264299 (PMC8906618; doi:10.1371/journal.pone.0264299)
Supplement: Attachment — Submitted filename: rev3 - response_for_editor_K9.pdf [file pone.0264299.s005.pdf]

RESPONSE TO EDITOR

on the article titled

Development of a detailed canine gait analysis method for evaluating harnesses: a pilot study

submitted to the peer-reviewed journal

PLOS ONE

Dear Dr. Ibañez,

We have received your comment regarding our article! Based on your comment we have revised our manuscript, and we hope that our revisions prove to be satisfactory! Thank you for your insightful critique.

1. *Please amend your Response to Reviewers letter to include a point by point response to each of the points made by the Editor and / or Reviewers. Please follow this link for more information: http://blogs.PLOS.org/everyone/2011/05/10/how-to-submit-your-revised-manuscript/*

   Thank you for the comment. The created point-by-point responses to the Editor (Dr. Ewa Tomaszewska) and the Reviewer (Nicola Francesco Lopomo) have been attached again in the revision. We have indicated in the file name (with *'Rev2'* prefix) that these belong to the second revision round. We also attached this second Response to the Editor letter (with '*Rev3*' prefix) to reflect the upcoming issues during the submission checking process.

2. *We note that the grant information you provided in the 'Funding Information' and 'Financial Disclosure' sections do not match.*

   Thank you for bringing our attention to this point! The research itself was supported by the National Research, Development and Innovation Fund (TKP2020 IES, Grant No. BME-IE-BIO and No. BME-NC). However, the publication is funded by the Hungarian Scientific Research Fund (OTKA). The reason why the two OTKA grant number do not match in the two mentioned section is because at that time when this paper was submitted to the PLOS journal the old OTKA research fund (grant number: K115894) should have been charged for the publication. Since this research fund ended in July 2021, the new research fund (grant number: K135042) will be charged as soon as the manuscript is accepted. During the latest revision we were only able to change the 'Funding Infromation' section. We assumed that the 'Fincancial Disclosure' can only be changed once (during the first submission).

   Finally, we would like to update the Funding Statement as follows:

> The research reported in this paper and carried out at BME has been supported by the Hungarian Scientific Research Fund (OTKA), grant number: K135042 (http://nyilvanos.otka-palyazat.hu/index.php?menuid=930&num=135042&lang=EN), and the National Research, Development and Innovation Fund (TKP2020 IES, Grant No. BME-IE-BIO and No. BME-NC) based on the charter of bolster issued by the National Research, Development and Innovation Office under the auspices of the Ministry for Innovation and Technology, Hungary. The three harnesses from Julius-K9® was provided free of charge by the manufacturer. The funders had no role in study design, data collection and analysis, decision to publish, or preparation of the manuscript.

3. *Thank you for stating the following in the Acknowledgments Section of your manuscript:*

"The research reported in this paper and carried out at BME has been supported by the Hungarian Scientific Research Fund (OTKA), grant number: K135042, and the National Research, Development and Innovation Fund (TKP2020 IES, Grant No. BME-IE-BIO and No. BME-NC) based on the charter of bolster issued by the National Research, Development and Innovation Office under the auspices of the Ministry for Innovation and Technology, Hungary. "

We note that you have provided funding information that is not currently declared in your Funding Statement. However, funding information should not appear in the Acknowledgments section or other areas of your manuscript. We will only publish funding information present in the Funding Statement section of the online submission form. Please remove any funding-related text from the manuscript and let us know how you would like to update your Funding Statement.

Thank you for the insightful comment. The Acknowledgement has been revised and all the funding-related text have been removed. The new chapter reads as follow:

> **Acknowledgments**
>
> The authors would like to thank Dr. Otília Biksi for her contribution in overseeing the experiments.

Finally, we would like to update the Funding Statement as follows:

> The research reported in this paper and carried out at BME has been supported by the Hungarian Scientific Research Fund (OTKA), grant number: K135042 (http://nyilvanos.otka-palyazat.hu/index.php?menuid=930&num=135042&lang=EN), and the National Research, Development and Innovation Fund (TKP2020 IES, Grant No. BME-IE-BIO and No. BME-NC) based on the charter of bolster issued by the National Research, Development and Innovation Office under the auspices of the Ministry for Innovation and Technology, Hungary. The three harnesses from Julius-K9® was provided free of charge by the manufacturer. The funders had no role in study design, data collection and analysis, decision to publish, or preparation of the manuscript.

Best regards:

Zsófia Pálya, Kristóf Rácz, Gergely Nagymáté & Rita M. Kiss
